# Novel Sulfur‐Rich Polymers from Inverse Vulcanization as Functional Building Blocks for Photonics

**DOI:** 10.1002/marc.202500957

**Published:** 2026-02-08

**Authors:** Raimondo Insogna, Fabiano Martorelli, Daniela Di Fonzo, Martina Martusciello, Roberto Utzeri, Angelo Angelini, Davide Comoretto, Paola Stagnaro

**Affiliations:** ^1^ Istituto Di Scienze e Tecnologie Chimiche “Giulio Natta”, Consiglio Nazionale delle Ricerche SCITEC‐CNR Genova Italy; ^2^ Dipartimento di Chimica e Chimica Industriale Università degli Studi di Genova, DCCI‐UNIGE Genova Italy; ^3^ Istituto Nazionale di Ricerca Metrologica, INRiM Torino Italy

**Keywords:** all‐polymer photonic crystals, high‐refractive‐index polymers, inverse vulcanization, isopropenyl aromatics, metasurfaces, sulfur upcycling, Suzuki–Miyaura cross coupling reaction

## Abstract

Novel Inverse Vulcanized Polymers (IVPs) with high refractive index and excellent transparency in the NIR are achieved exploiting the recently emerged Inverse Vulcanization (IV) process as a simple, green, efficient, and scalable synthetic method to upcycle elemental Sulfur, which is accumulating in the environment as a by‐product of the oil and gas industry. Co‐ and ter‐polymers with Sulfur content 50 or 60 wt% are prepared in quantitative yields utilizing different crosslinking aromatic comonomers bearing isopropenyl functionalities. To the best of our knowledge, for the first time, two novel diisopropenyl aromatics, namely 2,7‐diisopropenylfluorene (DIF) and 2,8‐diisopropenyldibenzothiophene (DIDBT), are designed, prepared in very high yields via a sustainable, facile, single‐step Suzuki–Miyaura cross‐coupling reaction, and explored as suitable crosslinking comonomers for the IV reaction. Subsequently, comonomer(s) nature, number of their functionalities, as well as Sulfur‐to‐comonomer(s) ratio are varied to address both the optical behavior and filmability of the ensuing IVPs, which are technologically relevant properties for their application and industrial scale‐up. Crosslink density is nicely correlated with the experimental glass transition temperatures of the investigated systems. Synthesized IVPs are then employed to fabricate all‐polymer distributed Bragg reflectors (DBRs) and imprinted optical components. Results pave the way for diverse applications of IVPs in advanced nanophotonics.

## Graphical abstract

Isopropenyl aromatic comonomers, in particular novel 2,7‐diisopropenylfluorene and 2,8‐diisopropenyldibenzothiophene, synthesized by sustainable, facile, single‐step SMCCR from available brominated substrates are exploited in the Inverse Vulcanization process to obtain functional Sulfur‐rich macromolecular materials. Tunability of crosslink number and glass transition temperature, and hence of processability, by structure design makes these Sulfur‐rich materials promising building blocks for light‐emission‐control applications.

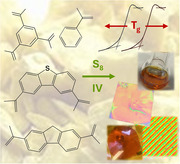

## 1 Introduction

Sulfur is one of the most abundant elements on Earth, commonly found in minerals from volcanic regions, it also plays a role as a common reaction site within biological systems. Nowadays Sulfur is no longer extracted from mines, since elemental Sulfur is produced ubiquitously and in huge quantities as a major by‐product of oil and gas refinery [1, 2, 3, 4]. Most of the Sulfur produced is transformed into sulfuric acid, which is extensively used as batteries component, in fertilizer manufacturing, and in the production of a variety of other chemicals, pharmaceutical compounds, (bio)materials for biological applications. With the aim of making waste Sulfur a resource, renewed interest has arisen in the last decades in Sulfur‐based reactions; thus, beside the great success of thiol‐click chemistry [5, 6], other keywords have grown‐up, such as, Lithium‐ and Sodium‐Sulfur batteries [7, 8], Sulfur polymers [3, 4, 9, 10, 11, 12].

In particular, amongst promising environmentally friendly uses of Sulfur, Inverse Vulcanization (IV) process has emerged as an efficient method for developing polymers with unique optical and mechanical properties [13, 14]. Unlike classical rubber vulcanization, where the elastomeric chains are crosslinked with minor amounts of Sulfur [15], in the IV process massive elemental Sulfur is crosslinked with minor‐to‐medium amounts of suitable unsaturated hydrocarbon comonomer(s), to give randomly crosslinked Sulfur‐rich polymeric architectures. The IV process takes advantage of Sulfur's tendency to open its eight‐atom‐ring molecule and form linear polysulfide chains at temperatures as high as 160°C–190°C (that is well above its melting temperature occurring around 115°C) to bind added divinyl molecules to the Sulfur radicals being formed. Typically, the crosslinking comonomers are aromatic hydrocarbons bearing divinyl (or analogous) [9, 13, 14] functionalities able to form bridges between the polysulfide chains, via an essentially radical mechanism [9, 16].

Main features of IV reaction offer various advantages and possibilities of exploitation:

- Molten elemental Sulfur acts as reactant and solvent, with no need for organic solvents [13];
- The reaction yields are practically quantitative, in compliance with the atom economy principle;
- For the above‐mentioned reasons, the process can be easily scaled up [17];
- Co‐ and ter‐polymers with very high content of Sulfur (even up to 90 wt%) can be obtained [9, 18];
- Diverse crosslinking unsaturated organic comonomer types, e.g., aromatic alkenes [19, 20, 21], alkynes [22, 23], cyclic olefins [18, 24], natural olefinic products [18, 25, 26, 27], aliphatic benzylic comonomers [28], as well as molecules bearing other functional groups [16, 29, 30] are suitable for the IV reaction to give a variety of novel macromolecular architectures;
- Selenium chalcogen chains can be introduced in the IV products as well [31, 32, 33];
- The crosslink density can be varied using comonomers at different number of reactive functional groups.

Due to their peculiar nature, functional properties of the IV products, christened Inverse Vulcanized Polymers (IVPs), are unique and quite intriguing as well:

- They are thermally stable up to about 220°C–260°C [21];
- Being structures with random connectivity between Sulfur chains and organic co‐units, they exhibit amorphous behavior, with resultant glass transition temperature (*T*_g_) depending on comonomer(s) nature, functionality, and content [18, 21];
- The low bonding energy of the S─S bond (266 kJ/mol, with respect to 347 kJ/mol for C─C) in the polysulfide chains, leads to dynamic covalent behavior [29, 32], that is IVP materials can be processed as thermoplastics and possess self‐healing capability;
- The high content of easily polarizable Sulfur atoms endows such materials with a very high refractive index over a broad spectral range coupled to good transparency in the near infrared region (NIR) [20, 21, 32, 33], which is in the telecommunication spectral window.

In this context, we develop the idea of obtaining—from waste Sulfur and through Inverse Vulcanization with suitable comonomers—novel Sulfur‐rich materials possessing high refractive index (*n*) and excellent NIR transparency. Our main scope was indeed to exploit the IV process as a simple, efficient, and promising method of upcycling Sulfur in the design and development of new high‐added‐value functional polymeric materials suitable as high‐*n* building blocks in the fabrication of all‐polymer 1D planar photonic crystals (PhCs) and nanoimprinted metasurfaces.

All‐polymer PhCs, i.e., nanostructures with sub‐micrometric modulation of the refractive index, with respect to the—so far—more performing inorganic counterparts, present several advantages, such as: materials affordability and low processing costs; scalability and opportunity of covering large areas; light weight; mechanical flexibility; use of upcycled materials and possibility of obtaining free‐standing structures [34, 35]; permeability to small molecules both in the vapor and liquid phase as well as the consequent swelling allow for label‐free photonic sensors [35, 36]. All these features can open new strategies for technological applications in the field of photonic, optical, sensing, and metamaterial devices.

However, the application of all‐polymer photonic structures in the field of light confinement is limited by the intrinsically low and similar refractive indices typical of polymers (*n* = 1.5‐1.6) in their transparency window [37]. The refractive index contrast (*Δn*), in fact, plays a key role in the light confinement: the larger Δn, the more efficient this effect. This results in higher reflectance intensity and larger spectral width of the resultant photonic band gap (PBG); in other words, fewer layers are necessary for achieving high reflectivity, with consequent fabrication shortening and material saving [37]. Additionally, the fabrication of all‐polymer PhCs requires the mutual processability of the two (high‐*n* and low‐*n*) dielectric components, further limiting the choice of suitable pairs and in turn reducing the dielectric contrast achievable in the transparency region of the polymers. In this respect, the peculiar features of IVPs, make them appealing building blocks to develop new polymeric materials for optical applications in the field of emerging key enabling technologies, such as quantum photonics, non‐linear optics, and metamaterials.

In previous studies, in order to achieve IVPs exploitable for photonic structures fabrication, a novel comonomer for the IV process, namely 2,5‐diisopropenylthiophene (**DIT**), was purposely designed and first synthesized [38, 39, 40] from 2,5‐dibromothiophene substrate through a facile, environmentally friendly, single‐step Suzuki–Miyaura cross‐coupling reaction (SMCCR) [41, 42, 43, 44].

Methyl groups borne by the isopropenyl functionalities could ensure higher reactivity in the IV radical process with respect to vinyl counterparts, such as commonly used 1,3‐divinylbenzene. Moreover, the aromaticity of the π‐conjugated thiophene ring and the presence of the Sulfur atom itself might confer high electronic polarizability [45] with consequent increase of the refractive index of the ensuing IVP materials, as also observed in relevant recent works on other Sulfur‐rich macromolecular structures [46, 47, 48].

**DIT** comonomer was then successfully employed in the IV reaction, obtaining a series of random **S‐DIT** copolymers with Sulfur content ranging from 60 up to 90 wt.%. Such **DIT**‐based IVP systems exhibited a very high refractive index, with values around 1.85 at 600 nm, hat keep greater than or equal to 1.8 up to 2500 nm, thus covering the whole NIR region [38].

The **S‐DIT** copolymers revealed also an excellent transparency in the same wavelength range.

Despite the low, sub‐ambient *T*_g_s that partially hampered their filmability, the **S‐DIT** copolymers were blended with polyvinylcarbazole (PVK, *n* = 1.67 at 600 nm), a commercial high‐*n* polymer used in the fabrication of polymeric distributed Bragg reflectors (DBRs), also referred to as dielectric mirrors, to improve their processability. The obtained blends were employed as high‐n medium, which was alternated to polyacrylic acid (PAA, *n* = 1.51 at 600 nm), as the low‐*n* component. Good quality all‐polymer DBRs, that is highly transparent media with very high reflectance at the PBG, which are fundamental bricks for photonic structures, were obtained even with a limited number of layers [38, 40, 49].

**CHART 1:**
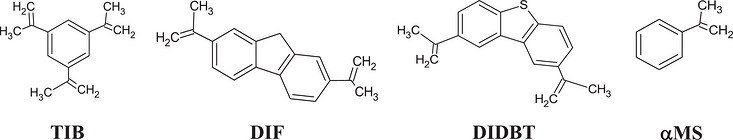
Isopropenyl aromatic derivatives used in this work as comonomers for the IV reaction.

In this work, in order to enhance processability of IVPs and modulate their glass transition by varying the crosslinking density, a series of co‐ and ter‐polymers was synthesized using the already reported trifunctional comonomer triisopropenylbenzene (**TIB**) [21] and commercial alpha‐methylstyrene (**αMS**), as monofunctional counterpart. Furthermore, two novel diisopropenyl condensed aromatic derivatives with a more extended π‐conjugation, namely 2,7‐diisopropenylfluorene (**DIF**) and 2,8‐diisopropenyldibenzothiophene (**DIDBT**) molecules, were for the first time designed, prepared, and used as suitable comonomers for the IV reaction (Chart 1). The achieved IVPs were characterized, and their *T*_g_ values correlated to the crosslink number and nature of the comonomer(s). Finally, the most processable IVPs were used to prepare multilayered dielectric mirrors as well as microimprinted diffraction gratings and Fresnel lenses as a first step toward metalenses.

## 2 Results and Discussion

As mentioned above, over the last dozen years, inverse vulcanization has attracted growing interest with a proliferation of suitable comonomers and multiple application opportunities for the so formed Sulfur‐rich polymer materials [31, 50, 51, 52].

Despite these encouraging beginnings [9, 51, 52], various issues related to IV reaction mechanism [16, 29, 53, 54, 55] and challenges in the effective application of the ensuing IVPs are still open [19, 56]. This process is indeed in an early stage of development as testified by the diverse experimental setups and conditions described in the literature [51, 56], such as, different reaction times, variable order of reagents addition, use or not of controlled atmosphere, process temperatures variable in a certain range, post‐reaction thermal curing treatments.

IVPs are not as simple to process as often claimed. Indeed, some issues that have emerged need to be addressed, taking into account the following aspects:

- Volatility of organic crosslinking comonomers, which may lead to actual copolymer compositions quite different from the nominal feed ratio [51];
- Stability of formed IVPs over time: due to a certain tendency to depolymerize, especially at high Sulfur content and high temperatures, releasing back elemental Sulfur, through the radical process of backbiting [31, 57];
- Poor solubility in suitable organic solvents (e.g., toluene) useful for subsequent treatments;
- Filmability (processability) hampered in case of *T*_g_ values too low (too fluid) or too high (excess of crosslinking, high viscosity, and then reduced solubility) [38, 58, 59].

All these issues are determinant for the foreseen applications and IVPs’ industrial scale‐up.

This work aims to meet this challenge: to make IVPs active key‐elements for photonics and metamaterials by chemical engineering of their electronic response, which in turn controls their refractive index. The double scope pursued is to exploit these high‐refractive‐index Sulfur‐based macromolecules as well as to control their crosslinking density to make them processable for fabrication of multilayered nanostructures or imprinted metasurfaces for photonics.

### 2.1 Synthesis of Isopropenyl Aromatic Comononers

Various trifunctional and difunctional isopropenyl aromatic derivatives, purposely synthesized in our laboratories, and commercial alpha‐methylstyrene (**αMS**) as monofunctional counterpart (Chart 1), were exploited as suitable comonomers for the IV process with the aim of modulating the crosslinking density and *T*_g_ and, consequently, possibly improving the processability and serviceability window of the ensuing IVP materials.

Specifically, 1,3,5‐triisopropenylbenzene (**TIB**), which is already described in the literature [21], and 2,7‐diisopropenylfluorene (**DIF**) and 2,8‐diisopropenyldibenzothiophene (**DIDBT**) comonomers, to the best of our knowledge for the first time here reported, were synthesized from the corresponding commercially available brominated substrates via the SMCCR protocol (see the Experimental Section) we previously optimized for preparing **DIT** comonomer [38, 40].

The two newly synthesized molecules **DIF** and **DIDBT**, which both are solid compounds with melting points of 212°C and 94°C, respectively, were obtained in very high yields (93% and 95%, respectively) and characterized by IR, ^1^H and ^13^C NMR spectroscopies to confirm their expected chemical structures (see the Experimental Section). Related spectra are collected from Figures S1–S6 (Supporting Information hereafter).

### 2.2 Synthesis and Main Characterization of IVPs

The conditions here adopted for the IV reaction of elemental Sulfur with the envisaged isopropenyl comonomers are detailed in the Experimental Section. In all cases, the expected random IVPs were obtained in practically quantitative yields.

The photographs reported in Figure S7 show the reaction mixture appearance during the initial stages (typically within 10 min) of the IV process and a typical glassy IVP product recovered at its end. It is also worth noticing here that, irrespective of their composition, the IVPs prepared in this work are quite stable upon ageing at ambient conditions, even after several months from their synthesis (as testified by the pictures in Figure S8), without showing any apparent de‐mixing of elemental Sulfur, due to possible backbiting of the polysulfide chains formed in‐between the hydrocarbon crosslinkers to give back the S_8_ molecule. In Figure S9, the chemical structures of the new random IVP copolymeric systems containing the newly synthesized **DIF** and **DIDBT** comonomers are sketched.

In order to enhance the crosslinking degree and hence reduce segmental chain mobility with consequent increase of *T*_g_ of the ensuing IVPs, our initial choice fell on the use of trifunctional **TIB** comonomer. Indeed, relatively high *T*_g_ values should allow for the fabrication of polymeric (multi)layers with good optical quality.

The **S‐TIB** copolymers were prepared in 50/50 and 60/40 wt/wt feed ratios in order to maintain a quite high content of Sulfur, which is worth remembering here is a major byproduct of the oil and gas industry that needs to be valorized into high‐value products.

Main information on the synthesized **TIB**‐based IVPs was acquired by infrared spectroscopy and thermal analyses.

**FIGURE 1:**
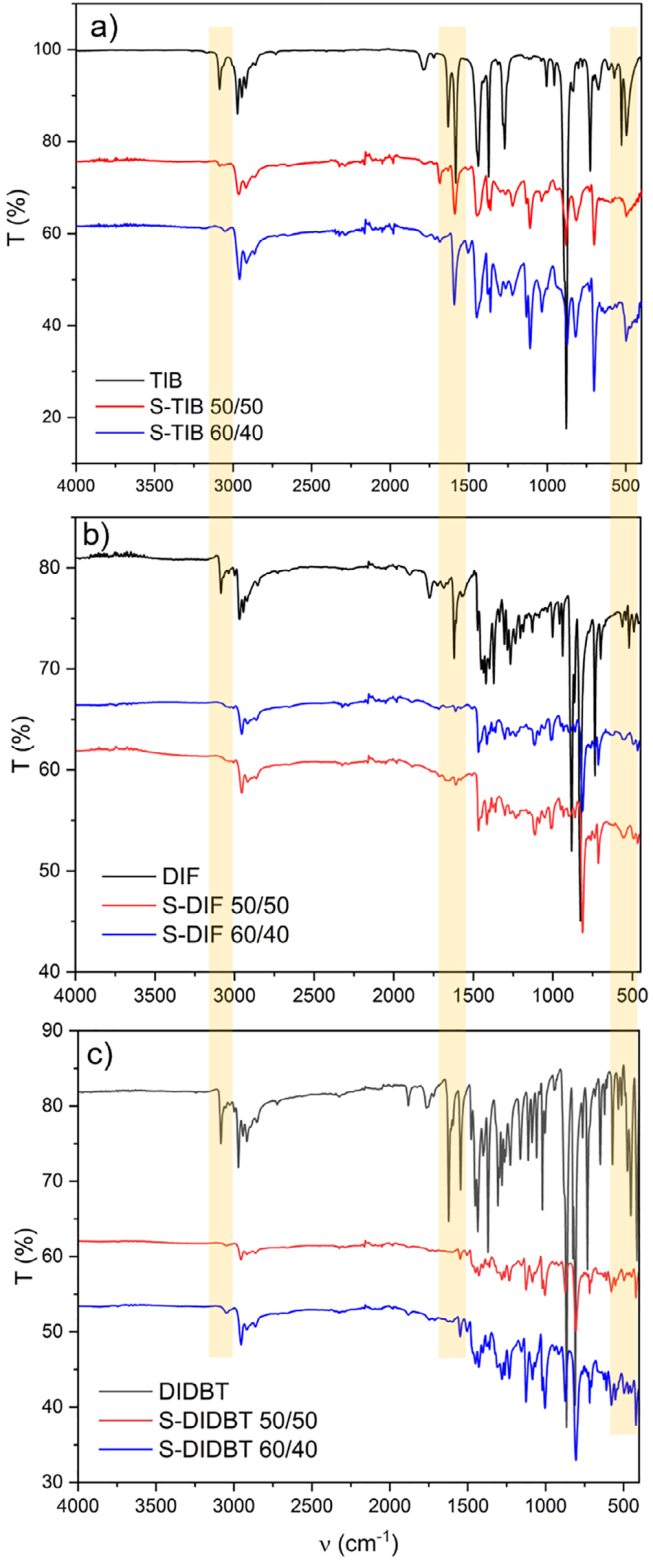
ATR‐FTIR transmittance spectra of: a) **TIB** comonomer and **S‐TIB** 50/50 and 60/40 wt/wt copolymers; b) **DIF** comonomer and **S‐DIF** 50/50 and 60/40 wt/wt copolymers; c) **DIDBT** comonomer and **S‐DIDBT** 50/50 and **S‐DIDBT** 60/40 wt/wt copolymers.

FTIR transmittance spectra, collected by Attenuated Total Reflectance (ATR), of the inverse vulcanization products gave evidence of the formation of the expected copolymeric structures (Figure 1a, yellow bands highlighting the most meaningful vibrational modes). As visible in the figure, the strong absorbance in the infrared spectrum of **TIB** monomer between 3100 and 3000 cm^−1^, due to the presence of both isopropenyl and aromatic = C─H stretching bands partially overlapped, drastically decreases in intensity upon IV reaction. By comparing the spectra of unreacted **TIB** with those of the two IV products, the narrow band at 3086 cm^−1^ of the isopropenyl = C─H stretching completely vanished, leaving only a weaker and broader signal centered at 3050 cm^−1^ due to the aromatic = C─H stretching. This was accompanied by the disappearance of the peak at 1630 cm^−1^, characteristic of C═C stretching of the isopropenyl moiety, and the maintenance of the aromatic C═C stretching at 1583 cm^−1^. The strong absorptions at 878 and 726 cm^−1^, ascribed to the out‐of‐plane bending of aromatic = C─H in 1,3,5‐trisubstituted benzene ring were retained as well, even though with modified intensity ratios. The stretching signals of the newly formed C─S and S─S bond expected in the copolymers’ spectra are usually very weak and typically fall in the 700–590 and 540–500 cm^−1^ region, respectively. In fact, some changes, more evident in the sample with 60 wt% Sulfur, could be detected in these regions without however being able to give a certain assignment for the two signals mentioned above.

Main data resulting from thermal analyses carried out by TGA and DSC on the **TIB**‐based IVP series are summarized in Table 1.

**TABLE 1:** TGA and DSC data of IVP series containing **TIB** and **αMS** comonomers.

Sample	*T*_Donset_ ^a^ [°C]	*T*_D5%_ ^b^ [°C]	*T*_Vmax_ ^c^ [°C]	*Δwt*_S_ ^d^ [%]	*Δwt*_C_ ^e^ [%]	*T*_g_ (I h)^f^ [°C]	*T*_g_ (II h)^g^ [°C]
S‐TIB 60/40	260	254	278	45	39	78^h^	73
S‐TIB 50/50	264	261	287	42	44	120	115
S‐TIB‐αMS 50/45/5	262	256	282	47	40	99	99
S‐TIB‐αMS 50/40/10	257	246	274	46	37	65	65
S‐TIB‐αMS 50/30/20	234	220	268	46	11	29	29
S‐TIB‐αMS 50/20/30	235	220	271	50	10	6	6

^a^ Corresponding to a weight loss of 1%–2%;

^b^ Corresponding to a weight loss of 5%;

^c^ Corresponding to the maximum rate of weight loss of the first degradation step;

^d^ Weight loss between onset of decomposition up to end of the first degradation step;

^e^ Weight loss between 700°C and 850°C;

^f^ Recorded on first heating (as prepared sample);

^g^ Recorded on second heating (after controlled cooling);

^h^ Broad endothermic phenomenon centered at about 112°C.

The **S‐TIB** copolymers were, as expected, characterized by higher *T*_g_ values compared to the sub‐ambient ones measured for the previously achieved **S‐DIT** copolymer series [40]; however, they revealed poor solubility (see further in the text) in key solvents, such as toluene.

For this reason, **TIB** was combined with the monofunctional **αMS** comonomer to tune the crosslinking density and hence *T*_g_ of the ensuing macromolecular structures. Random **S‐TIB‐αMS** terpolymers at increasing content of **αMS**, namely in 50/45/5, 50/40/10, 50/30/20, and 50/20/30 wt/wt/wt ratios were also prepared.

**FIGURE 2:**
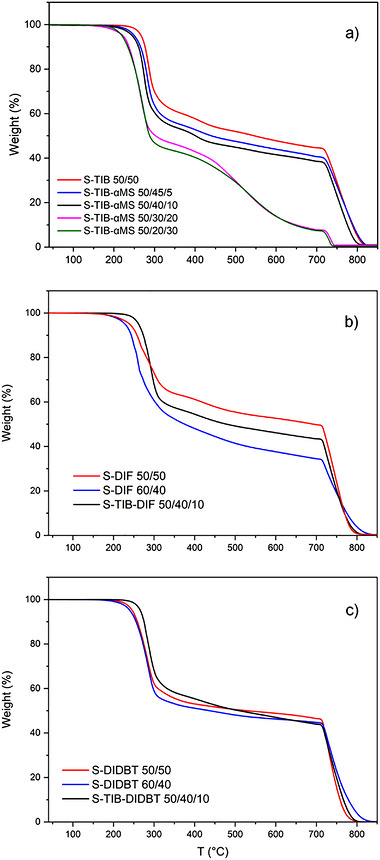
TGA curves for the IVP series: a) **S‐TIB‐αMS** containing 50 wt% Sulfur and increasing amounts of monofunctional **αMS** comonomer; b) **S‐DIF** 50/50 and 60/40 wt/wt and **S‐TIB‐DIF** 50/40/10 wt/wt/wt; c) **S‐DIDBT** 50/50 and 60/40 wt/wt and **S‐TIB‐DIDBT** 50/40/10 wt/wt/wt.

TGA measurements (Table 1 and Figure 2a) demonstrated that the nominal Sulfur/comonomer(s) ratios were maintained upon the IV reaction. The effective Sulfur and comonomer(s) contents were derived by the percentage of weight loss between onset of decomposition up to the end of the first degradation step (*Δwt*_S_%), essentially due to thermal degradation of polysulfide chains [40], and the percentage of weight loss between 700°C and 850°C (*Δwt*_C_ %), indicative of comonomer(s) carbonaceous residue combustion upon introduction of oxygen into the instrument furnace at 700°C.

Thermogravimetric analysis also revealed a relatively high thermal stability of the **TIB**‐based IVP series, with degradation onset temperatures (*T*_Donset_) around 235°C–265°C, temperatures at which the sample weight loss is 5% (*T*_D5%_) varying from 220°C to 260°C, and temperatures of maximum mass loss rate (*T*_Vmax_) for the first degradation step comprised in the range from 270°C and 290°C. These values are in line with those observed for pure Sulfur [40]. More in detail, for the terpolymers at relatively high content of monofunctional **αMS** comonomer (that is, reducing the total number of crosslinks in the resulting macromolecular structure), lower *T*_Donset_ values, around 235°C, were observed, together with a much lower carbonaceous residue.

DSC measurements (see Figures S10 and S11) revealed the expected amorphous behavior of the **TIB‐**based co‐ and terpolymers investigated, which exhibit only the glass transition, with the exception of the **S‐TIB** copolymer at 60/40 wt/wt ratio, whose first heating thermogram showed a weak endothermic phenomenon centered around 112°C. This could be assigned to small amount of unreacted Sulfur (perhaps due to incomplete mixing of the two reactants during IV reaction that produces a rapid increase in viscosity of the system) or, as already observed in the case of IVPs with high percentage of Sulfur [13, 40], to the melting of relatively long polysulfide chains connecting the hydrocarbon crosslinks. The DSC thermograms of the homogeneous **TIB**‐based series containing 50 wt% Sulfur exhibited decreasing *T*_g_ by increasing the content of monofunctional **αMS** in the IVPs composition (Table 1), with neither evident enthalpic relaxations at glass transition nor endothermic phenomena ascribable to melting of polysulfide chains during the first heating run.

**FIGURE 3:**
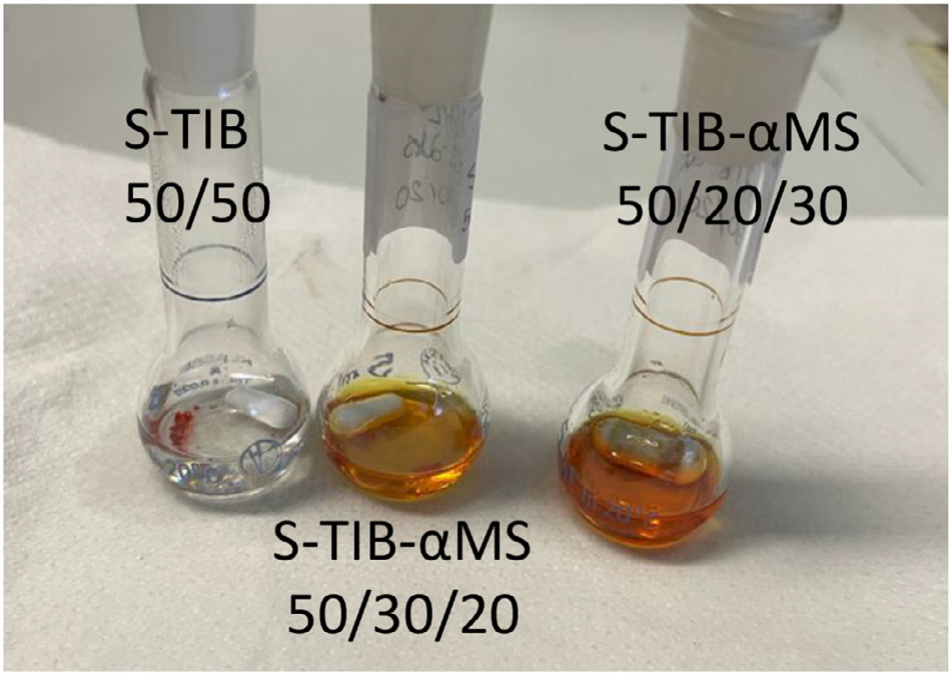
Solubility tests in toluene on IVP samples from the **TIB‐**based series.

Solubility tests in toluene (solvent of choice for the high‐*n* component in the fabrication of all‐polymer photonic structures, which are processed by spin casting) were carried out at concentration of 10 mg mL^−1^ by gently warming the systems at 60°C under magnetic stirring. Figure 3 highlights that the presence of increasing amounts **αMS** in addition to the **TIB** comonomer effectively improves the solubility of the IVPs, even though without achieving complete solubilization (especially at the even higher concentrations required to fabricate good optical quality DBRs), while, on the other hand, reducing the *T*_g_ values down to sub‐ambient temperatures.

On the basis of these not fully satisfying findings, but relaying on the promising results already obtained with the series of **S/DIT** copolymers [38, 40], the two newly synthesized molecules, namely 2,7‐diisopropenylfluorene, **DIF**, and 2,8‐disisopropenyldibenzothiophene, **DIDBT**, were for the first time exploited as suitable difunctional comonomers for the inverse vulcanization process, with the double aim of (i) achieving more adequate solubility in toluene, and (ii) possibly enhancing the refractive index, and more in general the optical response of the Sulfur‐rich macromolecular architecture being formed, due to the more extended π‐conjugation of the condensate aromatic rings present in the core of such crosslinkers.

Infrared analysis of the new series of **DIF** and **DIDBT**‐based IVPs confirmed formation of the expected polymeric structures. In Figure 1b, the IR spectra of the **S‐DIF** copolymers at 50 and 60 wt% Sulfur are compared with that recorded for the **DIF** comonomer. Both the well‐evident absorbance of the = C─H stretching at 3085 cm^−1^ and C═C stretching at 1619 cm^−1^ related to the isopropenyl moieties of the comonomer are no longer present in the copolymers’ spectra, indicating the completion of the IV reaction. The other features due to the fluorene moiety in the core of this crosslinker are maintained.

The infrared spectra of the two analogous **S‐DIDBT** copolymers are reported in Figure 1c. Comparing them to the spectrum of the **DIDBT** comonomer, reported as well, it is possible to observe that the IV reaction also in this case has gone to completion, as indicated by the disappearance of the absorption peaks of the = C─H and C═C stretching characteristic of the isopropenyl moieties, respectively at 3084 and 1622 cm^−1^, while the signals due to the dibenzothiophenic core remain, as expected.

As for the **S‐TIB** copolymers (Figure 1a), also in the IVPs prepared from **DIF** (Figure 1b) and **DIDBT** (Figure 1c), no signals clearly attributable to the newly formed C─S bonds and to the S─S bonds of the polysulfide chains were evidenced.

Thermal analyses (TGA and DSC) of the synthesized **DIF** and **DIDBT** based IVPs were carried out as well. In Table 2, the values of the main data obtained from TGA and DSC measurements are reported.

**TABLE 2:** TGA and DSC data of IVP series containing **DIF**, **DIDBT**, and **TIB** comonomers.

Sample	*T*_Donset_ ^a^ [°C]	*T*_D5%_ ^b^ [°C]	*T*_Vmax_ ^c^ [°C]	*Δwt*_S_ ^d^ [%]	*Δwt*_C_ ^e^ [%]	*T*_g_ (I h)^f^ [°C]	*T*_g_ (II h)^g^ [°C]
S/DIF 60/40	244	228	260	45	35	34^h^	39
S/DIF 50/50	244	242	274	37	48	50	61
S/TIB/DIF 50/40/10	273	261	287	42	44	105	103
S/DIDBT 60/40	251	233	285	49	41	33	32
S/DIDBT 50/50	252	238	285	41	46	68	66
S/TIB/DIDBT 50/40/10	263	266	286	42	45	103	99

^a^ Corresponding to a weight loss of 1%–2%;

^b^ Corresponding to a weight loss of 5%;

^c^ Corresponding to the maximum rate of weight loss of the first degradation step;

^d^ Weight loss between onset of decomposition up to end of the first degradation step;

^e^ Weight loss between 700°C and 850°C;

^f^ Recorded on first heating (as prepared sample);

^g^ Recorded on second heating (after controlled cooling);

^h^ Endothermic peak centered around 117°C.

TGA thermograms of the **S‐DIF** series (Figure 2b) and analogous **S‐DIDBT** series (Figure 2c) were characterized by the expected double step profile of degradation, featuring first the degradation of polysulfide chains followed by the slower decomposition of the hydrocarbon portion to form a carbonaceous residue then combusted by oxygen introduction. As evident from both the Table and Figures, the weight losses of the two steps roughly confirmed the IVPs nominal feed ratio.

DSC measurements (see Figures S12–S14) revealed the expected amorphous behavior of the **S‐DIF** and **S‐DIDBT** copolymers and of terpolymers containing also **TIB** (Figure S14). In Table 2 the *T*_g_ values are reported. At equal content in Sulfur (and **TIB** when present as comonomer) the IVPs behave similarly, being comparable the condensate ring structure of the two **DIF** and **DIDBT** comonomers. In particular, **S‐DIF** 60/40 system (Figure S12) on the first heating run (as prepared sample) shows an endothermic peak centered at about 117°C, likely preceded by a broad exotherm occurring soon after the glass transition, which is characterized by an enthalpic relaxation peak. This phenomenon can be ascribed to the presence of unreacted Sulfur. In the first heating curve (Figure S13) the **S‐DIDBT** copolymers exhibit a glass transition, with a quite evident enthalpic relaxation peak, especially for the 50/50 wt/wt composition sample, and a tiny broad endothermal phenomenon in the range 85°C–95°C and 90°C–100°C, respectively, for **S‐DIDBT** 60/40 and 50/50 wt/wt, ascribable to melting of the polysulfide chains.

Solubility tests (Figure S15) on these newly synthesized IVPs series, evidenced that the **S‐DIF** 50/50 copolymer gave the best compromise between solubility in toluene and *T*_g_ (61°C).

**FIGURE 4:**
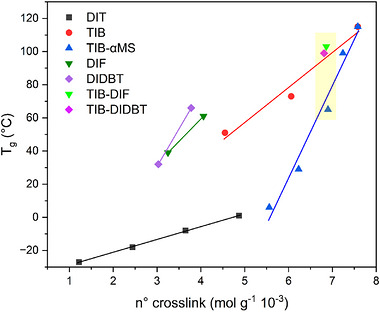
*T*_g_ values plotted vs crosslink number for the different IVP series listed in Table 3.

Table 3 below reports the experimental *T*_g_ values plotted against the number of crosslinks (in mol g^−1^ 10^−3^) for all the IVPs prepared in this and in a previous work [40]. Interestingly, each homogeneous series of copolymers shows a nice linear trend between the number of crosslinks and the measured *T*_g_. Even though there is still a lively debate on the mechanism of IV reaction [16, 29, 53, 54, 55], whereby each C═C double bond (also depending on its specific chemical surroundings) could lead to a different macromolecular network density, this does not affect this work. In fact, the proportion is here maintained because all comonomers considered have the same type of reactive functionality (that is the isopropenyl moiety) toward Sulfur. By observing the plots in Figure 4, several inferences can be drawn. For the same number of functionalities, e.g., 2, and number of crosslinks, the *T*_g_ values can change significantly because the aromatic core of the comonomer is of a different nature and therefore confers different intrinsic rigidity to the ensuing macromolecular structure. **DIDBT** and **DIF** comonomers, which possess a similar chemical structure differing for the presence of a Sulfur heteroatom (**DIDBT**) in the 5‐atom central ring in place of a methylene (**DIF**), exhibit indeed similar glass transition values and similar incremental growth, by varying the Sulfur content in the copolymer. Instead, the IVP series based on the smaller **DIT** comonomer [40], is characterized by much lower *T*_g_ values, which nicely fit a straight line with a slow slope. The number of crosslinks in Table 3 is given in millimoles per gram, but it should be noted that the compositions of IVPs are given by weight, so in a 50 wt% Sulfur copolymer, there will be more crosslinks if the comonomer has a lower molecular mass (*M*), obviously at an equal number of functionalities.

**TABLE 3:** Experimental *T*_g_ values versus calculated crosslink number for IVPs at different content of isopropenyl comonomers with different number of functionalities.

Sample	*Crosslink number* ^a^ [mol g^−1^ 10^−3^]	*T*_g_ ^b^ [°C]
S‐TIB 50/50	7.58	115
S‐TIB 60/40	6.06	73
S‐TIB 70/30	4.55	51^c^
S‐TIB‐αMS 50/45/5	7.24	99
S‐TIB‐αMS 50/40/10	6.90	65
S‐TIB‐αMS 50/30/20	6.23	29
S‐TIB‐αMS 50/20/30	5.56	6
S‐DIF 50/50	4.06	61
S‐DIF 60/40	3.25	39
S‐TIB‐DIF 50/40/10	6.86	103
S‐DIDBT 50/50	3.78	66
S‐DIDBT 60/40	3.03	32
S‐TIB‐DIDBT 50/40/10	6.81	99
S‐DIT 60/40	4.87	1^d^
S‐DIT 70/30	3.65	−8^d^
S‐DIT 80/20	2.44	−18^d^
S‐DIT 90/10	1.22	−27^d^

^a^ Calculated by considering the different functionalities of the employed comonomers: that is, 3 for **TIB**, 2 for **DIT**, **DIF**, and **DIDBT**, and 1 for **αMS**;

^b^ Recorded on DSC second heating (after controlled cooling);

^c^ From ref. [21];

^d^ From ref. [40].

Looking again at Figure 4, by comparing the two IVPs series containing the trifunctional **TIB** and difunctional **DIT** comonomer, one can observe two different linear trends. The **S‐TIB** copolymers (red symbols) show a slope higher than that evidenced for the **DIT** based counterpart (black symbols), the main difference being in this case the different number of isopropenyl reactive functions, 3 or 2, on an aromatic (**TIB**) or heteroaromatic (**DIT**) single‐ring core.

As for the **S‐TIB‐αMS** terpolymer series, the introduction of the monofunctional alpha‐methylstyrene, while keeping constant the Sulfur content at 50 wt%, results in a steep decrease of the glass transition temperatures. The *T*_g_ values of the three terpolymer samples: **S‐TIB‐DIF**, **S‐TIB‐DIDBT**, **S‐TIB‐αMS** at equal composition 50/40/10 wt/wt/wt, evidenced in the plot by the yellowish shaded area and differing only for the third comonomer nature and functionality, confirm the above reasoning. The introduction in the **S‐TIB** 50/50 system (*T*_g_ 115°C) of 10 percent by weight of the difunctional comonomers **DIF** or **DIDBT**, similar in their chemical structure and *M*, leads to similar crosslink numbers and lowers the *T*_g_ of about 15°C (see also Table 3). The same content by weight of monofunctional **αMS** produces instead a *T*_g_ drop of 50°C, being the *M* of **αMS** about a half with respect to those of the two difunctional comonomers the resultant crosslink number does not differ much from that of the other two terpolymers, but the smaller, less constrained molecules of **αMS** make the macromolecular structure much less rigid accounting for the great effect on *T*_g_ lowering.

Data reported above demonstrate not only the possibility of tuning the glass transition by varying the percentage of Sulfur content, but also by structural design of the IVPs, that is by varying the comonomer nature and/or functionality. Macromolecular parameters are indeed relevant for tuning the crosslink density, processability, and serviceability of the ensuing IVP systems [58, 59].

### 2.3 Growth of Dielectric Mirrors and their Optical Analysis

The preparation of the distributed Bragg reflectors requires the use of very high concentration polymer solutions (tens of milligrams per mL). Synthesized IVPs are still far from such solubility values. However, with respect to our previous findings [38], no de‐mixing effects are now observed due to the improved control of crosslinking. One problem still to be faced is the intrinsic evolution of their behavior over time. Indeed, the reconfigurable nature of the Sulfur‐Sulfur bonds, makes metastable the system reducing the solubility over time without significantly impacting on the thermal properties. In spite of that, here we report our findings in terms of optical properties.

**FIGURE 5:**
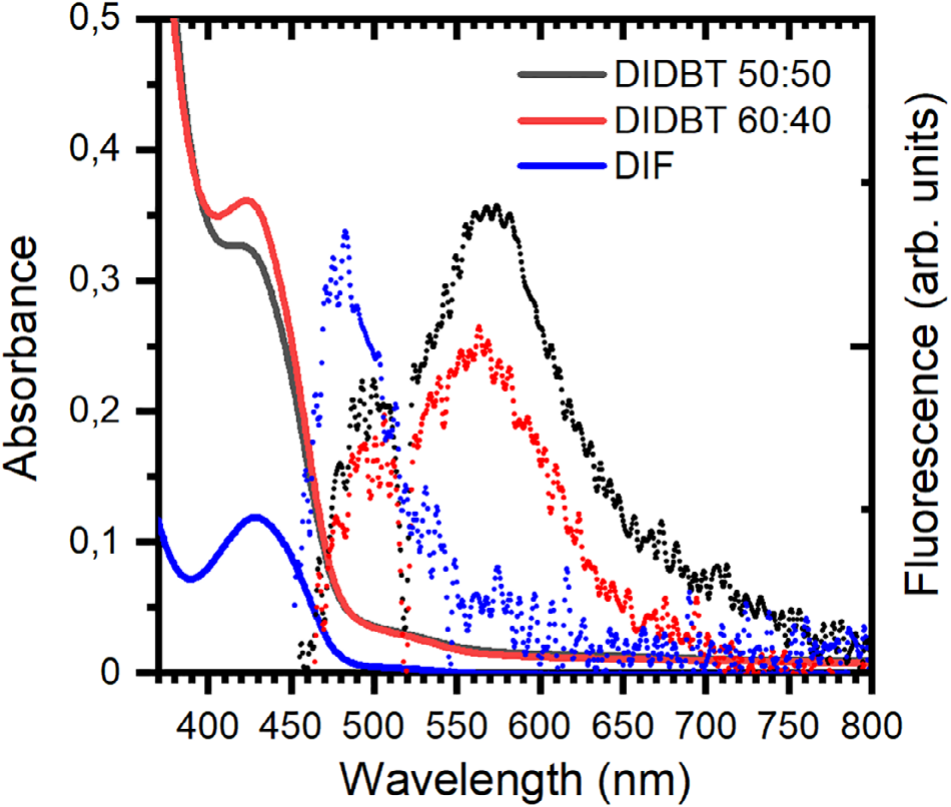
Absorbance (left scale) and fluorescence (right scale) spectra of **DIDBT** and **DIF** IVPs in toluene solution.

Figure 5 shows the absorbance spectra of IVPs containing **DIDBT** and **DIF** as crosslinking comonomers, in toluene solutions (<0.2 mg mL^−1^, see the Experimental Section) for few compositions. **DIDBT**‐based systems show an absorption onset at about 560 nm with a weak and broad feature up to 470 nm where a clear absorption rises with a peak at 423 nm. The latter is better resolved for the sample with higher sulfur content. For **DIF** based sample, the absorption structure is similar, and the peak is observed at 428 nm. Below 400 nm, a strong unresolved absorption where the solvent also plays a significant role, is observed. The overall similarity of the spectra in this spectral region for the two IVPs is assigned to the crosslinked polysulfide chains and minor differences in the nature of the crosslinkers.

Remarkably, IVP solutions show a weak but clear fluorescence presumably due to the nature of the crosslinker core moieties (Figure 5). **DIDBT** systems show a broad fluorescence peak at ∼570 nm with a satellite at ∼500 nm. For **DIF**, the fluorescence is mainly observed at ∼480 nm. The presence of the Sulfur heteroatom in the condensed aromatic molecule of **DIDBT** drastically modifies the fluorescence spectrum with respect to the fully hydrocarbon **DIF** fluorene derivatives, resulting in a bathochromic shift of about 90 nm. A similar effect is well known in the corresponding conjugated polymers where the absorption of polyfluorene is observed at higher energies with respect to dibenzothiophene based polymers [60, 61]. Figure S16 shows the fluorescence spectra of IVPs solutions recorded immediately after their synthesis. We noticed a higher solubility (<10 mg mL^−1^) allowing to record more intense fluorescence signal from **S**‐**DIDBT**, **S‐DIF** copolymers and their terpolymers containing also **TIB** with spectral features like the ones previously described.

**FIGURE 6:**
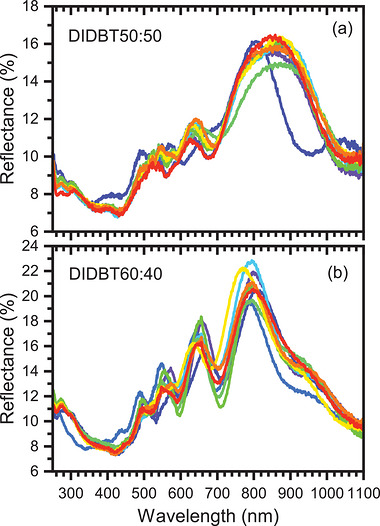
Reflectance spectra (9 spots) of (IVP:PS)‐CA DBRs made of 5.5 bilayers, where the IVPs were **S‐DIDBT** 50/50 (a) and 60/40 (b) copolymers.

Selected samples from these novel IVPs series were then used to prepare photonic structures which demonstrate the interesting optical behavior of such Sulfur‐rich materials, possibly exploitable for advanced photonic applications. As the procedure for DBRs preparation is very complex and requires a lot of time after the synthesis of the IVPs, we are forced to play with a material having lower solubility than the fresh one. For this reason, according to our previous findings, IVP polymers alone cannot be used to prepare the DBR [38]. However, they can be blended with different commodity polymers in order to achieve a suitable processability from solution. In this way, the refractive index of the blend depends on the volume fraction of the two components through the effective medium theory [62] while its direct determination for thin films is still challenging (see SI). When the amount of IVP is low, good quality DBRs can be effectively grown but their properties are mainly steered by the host polymer (usually polyvinylcarbazole or polystyrene, see Figure S17). Upon increasing the amount of IVP (solutions described in Figure 5), we have been able to prepare 5.5 bilayers DBR for two different copolymer compositions. Figure 6 shows the spectra of 5.5 bilayers DBRs grown with different Sulfur:**DIDBT** compositions blended in polystyrene. When Sulfur and DIBT have the same concentration (Figure 6a), a clear photonic band gap is observed at about 850 nm with a wider full width half maximum with respect to DBR grown with lower about of **DIDBT** (see Figure S17). Reflectance spectra recorded in different position on sample surface are quite homogeneous. The spectra background is dominated by interference fringes indicating a reasonable control of the overall sample thickness. Upon increasing the content of Sulfur (Figure 6b) the reflectance maximum is shifted at about 800 nm indicating a reduced films thickness. For both samples, the intensity is relatively weak probably due to light scattering induced by inhomogeneities within the films. In our case, the solubility is not still perfectly fitting the requirement of the growth thus limiting the processability and, consequently, the optical quality of the ensuing photonic structure. The relatively weak intensity of the peak reflectance observed in Figure 6, is assigned to the inhomogeneities in the film thickness due to the not‐ideal processability of the IVP:PS blend solution. This induces light scattering and reduced specular reflectance of the spin‐cast films making the measured reflectivity lower than that observed for DBR of a comparable number of bilayers made with commodity polymers or IVPs with larger refractive index [63, 64].

### 2.4 Replica Molding of IVPs Optical Components

In addition to filmability, IVPs are suitable for fabricating more complex optical components in a replica molding fabrication scheme (see details in the Experimental Section), which allows cheap and high throughput production of micro‐ and nano‐patterned devices.

It is worth noting that the solubility issues previously discussed do not affect the replica molding process, which concerns only the bulk properties. In fact, the reduced solubility observed after a long time following IVP synthesis becomes an advantage here, as it enhances structural stability and extends shelf life.

**FIGURE 7:**
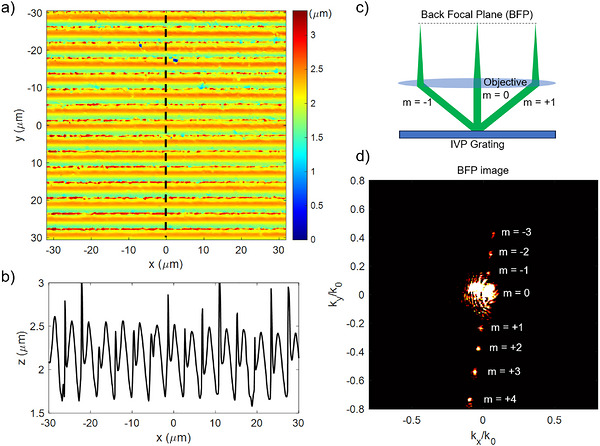
IVP Diffraction Grating fabricated by replica molding. a) Optical profilometer image of the diffraction grating (period 4 µm) obtained from S‐TIB‐DIF 50/40/10 wt/wt/wt terpolymer replica molding. b) Depth profile extracted along the dashed line from a). c) Schematic view of the BFP imaging. d) BFP image of the IVP diffraction grating showing the collected diffraction beams.

Figure 7a reports a false color image of the surface topography of a diffraction grating replicated in IVP as measured by an optical profilometer. The profile extracted along the black dashed line and reported in Figure 7b shows a depth modulation of about 1 µm. We assessed the ability of the IVP microstructure to diffract light in a Back Focal Plane (BFP) imaging scheme (see Figure 7c). Figure 7d shows that a collimated beam of visible light (*λ* = 520 nm) is effectively diffracted and several diffraction orders, collected by a 100x objective (N.A. = 0.8), are clearly visible. We notice a weak asymmetry of the intensity of the diffraction pattern assigned to a small curvature of the IVP grating.

**FIGURE 8:**
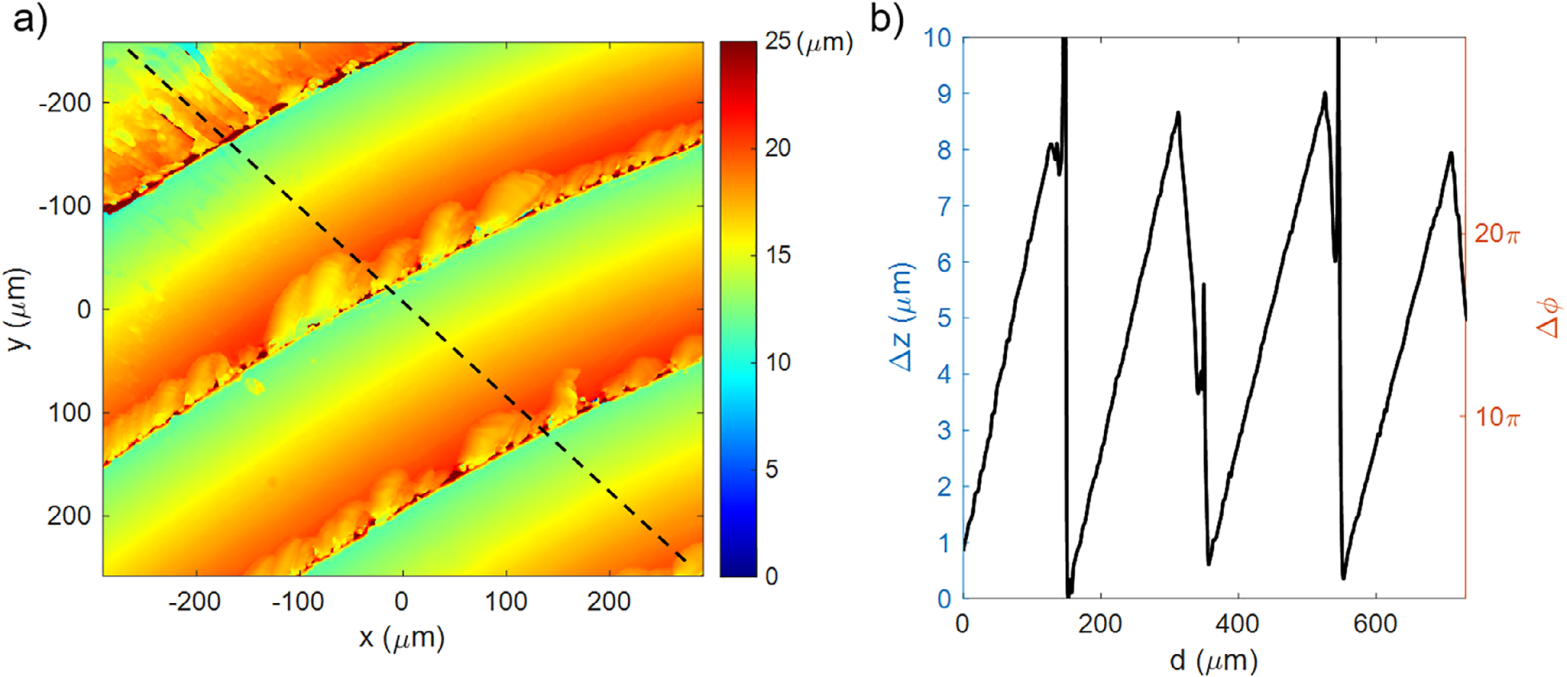
Fresnel lens portion fabricated by replica molding. a) Optical profilometer image of the diffraction grating (period 180 µm) obtained from S‐DIDBT 60/40 copolymer replica molding. b) Depth profile extracted along the black dashed line in a) and the corresponding phase profile calculated for λ = 520 nm (the scale of the two vertical axes has been normalized in order the two signals to be overlapped).

In addition to replica molding of microstructures, smooth profiles can also be used to template IVPs. Figure 8a shows the optical profilometer image of the IVP replica of a Fresnel lens. The depth profile shows a modulation depth as high as 8 µm, corresponding to a phase modulation depth of >20π (Figure 8b), calculated according to the following relation:

(1) $$\Delta \Phi =\frac{2\pi }{\lambda }\;\Delta z\left(n_{IVP}-1\right)$$

where *λ* = 520 nm and *n*_IVP_ = 1.85 (at 600 nm) [38, 40].

Here in particular, metasurfaces obtained by replica molding of IVPs, which gave well‐defined diffraction grating and Fresnel lens microstructures are shown. For optical metamaterials (and metasurfaces) a nanoscale downsizing will be necessary, but the results reported here represent the first step in this direction

## 3 Conclusions

Novel Sulfur‐rich IVPs were successfully synthesized exploiting the recently emerged IV process as an efficient method to upcycle waste Sulfur and achieve new functional materials with unique features.

As suitable crosslinking comonomers for the polysulfide chains, several isopropenyl aromatic derivatives were purposely designed and prepared via a facile single step SMCCR procedure.

Comonomer type and amount were tuned to enhance both the optical behavior and filmability of the resultant IVPs, that are the technologically relevant properties for their exploitation and industrial scale‐up.

For instance, the de‐mixing problem previously observed is now resolved. Moreover, for the first time, fluorescence emission has been demonstrated by IVPs. Distributed Bragg Reflectors have been grown by blending different amounts of IVPs with PS and using cellulose acetate as low dielectric material. DBRs show relatively good surface homogeneity and wide photonic band gap suggesting the IVPs slightly increase the refractive index of the host PS matrix. Replica molding has been also demonstrated as a suitable method to imprint into IVPs diffraction grating pattern as well as microstructured Fresnel lenses, thus paving the way toward nanoimprinted metamaterials for advanced photonics.

Open challenges in the field of polymer nanophotonics based on IVPs concern a further increase in solubility and an improved engineering of the macromolecular structures to limit the reconfigurable dynamic covalent S─S reaction, which leads to undesired back‐biting phenomena, and increase shelf‐life of photonic devices. This also would help in allowing a down‐scaling of the metastructures at sub‐micrometric level to fully exploit the potentiality of these high refractive index materials, while keeping the advantage of an easy and cost‐effective fabrication process.

## 4 Experimental Section

### 4.1 Chemicals and Materials

Elemental Sulfur in powder (S, Ph. Eur., BP), 1,3,5‐tribromobenzene (TBrB, 98%), alpha‐methylstyrene (**αMS**, *M* 118 g mol^−1^, 99%, contains 15 ppm *p*‐*tert*‐butylcatechol as inhibitor), 2,7‐dibromofluorene (DBrF, 97%), 2,8‐dibromodibenzothiophene (DBrDBT 97%), 4,4,5,5‐tetramethyl‐2‐(prop‐1‐en‐2‐yl)‐1,3,2‐dioxaborolane (isopropenylboronic acid pinacol ester, 95%, containing phenothiazine as stabilizer), tetrakis(triphenylphosphine)palladium(0) (Pd(PPh_3_)_4_, 99%), 1,4‐dioxane (ACS reagent, ≥99.0%), KOH (85%–100.5%), dichloromethane (DCM, ACS reagent, ≥99.5%,), n‐hexane (≥99%,), toluene (99.8%), 4‐hydroxy‐4‐methylpentan‐2‐one (98%), Na_2_SO_4_ (ACS reagent ≥99.0%, anhydrous, powder), silica gel (high‐purity grade, average pore size 60Å, 70–230 mesh, 63–200 µm, for column chromatography), polystyrene (PS, *M*_w_ = 192 000 g mol^−1^), cellulose acetate (CA, *M*_w_ = 50 000 g mol^−1^) were purchased from Merck‐Sigma‐Aldrich (Italy) and used as received. Air and/or moisture sensitive materials were manipulated under an inert atmosphere using dual vacuum/Argon lines.

### 4.2 Synthesis of Isopropenyl Comonomers by SMCCR

In a three‐neck flask, equipped with a magnetic stirrer and a bubble condenser, typically 1 g of the starting substrate (bromo‐substituted derivative), tetrakis(triphenylphosphine)palladium catalyst (1.5% eq. with respect to each Br), and potassium hydroxide (KOH, 2 eq. with respect to each Br) are introduced. The flask is kept under a gentle Argon flow. Meanwhile, in a separate two‐neck flask, a mixture of 1,4‐dioxane and water in a 2:1 ratio is prepared. A septum is placed on one of the necks to allow solvent (in proper amount to have 0.2 m substrate concentration) withdrawal via syringe, ensuring that the Argon flow remains uninterrupted throughout the system. Once the solvent mixture has been transferred, the proper amount (1.5 eq with respect to each Br) of isopropenylboronic acid pinacol ester reactant is added via syringe into the reaction flask. After these steps, the reaction flask is placed in an oil bath pre‐heated to 90°C and maintained under reflux for 24 h (the reaction completion was checked by TLC). The obtained reaction mixture is diluted with water and extracted four times with DCM. The combined organic phases are then transferred back into the separatory funnel and washed three times with water. Further washing is repeated three times with a saturated NaCl aqueous solution. The collected organic phase is then dried with anhydrous Na_2_SO_4_ over 24 h. The drying agent is then filtered off, and the filtrated solution brought to small volume using a rotary evaporator. The isopropenyl monomer product is purified by chromatography through a column packed with silica and using a mixture of 9:1 *n*‐hexane/DCM as eluent. In this way, starting from TBrB, DBrDBT, DBrF as substrates, the comonomers 1,3,5‐triisopropenylbenzene (**TIB**), 2,7‐diisopropenylfluorene (**DIF**) and 2,8‐diisopropenyldibenzothiophene (**DIDBT**) are respectively achieved in high purity and used as such for subsequent IV reaction. It is worth noting that the SMCCR procedure here used was successfully scaled up, starting from up to 5 g of bromo‐substituted substrate to achieve higher quantities of comonomer when necessary.

In the following, the main spectroscopic features of the synthesized comonomers are reported.

**1,3,5‐tri(prop‐1‐en‐2‐yl)‐benzene (TIB)**. *M* 198 g mol^−1^. Colorless liquid at room temperature. Actual yield 0.631 g. Yield 72%.

ATR‐FTIR: *ν* = 3086 (m; vinyl = C─H stretch, and w shoulder extending to 3030; aromatic = C─H stretch), 2972, 2945, 2919 (aliphatic ─C─H stretch), 1787 (w; overtone of aromatic C═C stretch), 1630 (m; vinylic C═C stretch), 1583 (m; aromatic C═C stretch), 878 and 726 (s and m; deformation of aromatic = C─H typical of 1,3,5‐trisubstituted benzene ring) cm^−1^.

^1^H NMR (400 MHz, CDCl_3_, δ): 7.46 (s, 3H, benzene ring CH), 5.38 (app quint, 3H, *J*_long range_ = −0.8 Hz, vinyl CH trans to CH_3_), 5.12 (app quint, 3H, *J*_long range_ = −1.5 Hz, vinyl CH cis to CH_3_), 2.19 (dd, 9H, *J*_long range_ = −1.5 Hz and *J*_long range_ = −0.8 Hz, CH_3_) ppm.

**2,7‐di(prop‐1‐en‐2‐yl)‐fluorene (DIF)**. *M* 246 g mol^−1^. Pale yellow solid, kk 190°C, mp 212°C. Actual yield 0.711 g. Yield 93%.

ATR‐FTIR: *ν* = 3085 (m; vinyl = C─H stretch, and w shoulder extending to 3030; aromatic = C─H stretch), 2967, 2944, 2921 (m; aliphatic ─C─H stretch), 1776 (w; overtone of aromatic C═C stretch), 1619 (vinylic C═C stretch), 1570 (aromatic C═C stretch), 1300–1000 (bending of the aromatic ring C─H), 881 and 826 (s; deformation of aromatic = C─H) cm^−1^.

^1^H NMR (400 MHz, CDCl_3_): δ = 7.71 (d, 2H, *J*_ortho_ = 8.0 Hz, C(4,5)H), 7.65 (app s, 2H, C(1,8)H), 7.50 (dd, 2H, *J*_ortho_ = 8.0 Hz and *J*_meta_ = 1.4 Hz, C(3,6)H), 5.43 (app s, 2H, C(11,14)H trans to CH_3_), 5.10 (app t, 2H, *J*_long range_ = −1.6 Hz, C(11,14)H cis to CH_3_), 3.90 (s, 2H, C(9)H_2_), 2.21 (app t, 6H, *J*_long range_ = −1.6 Hz, C(12,15)H_3_) ppm.

^13^C NMR (400 MHz, CDCl_3_): δ = 143.72 (2C, = C(10,13)‐CH_3_ quaternary), 143.55 (2C, C(2,7)H), 140.93 (2C, C(8a,9a) quaternary), 139.91 (2C, C(4a,4b) quaternary), 124.48 (2C, C(4,5)H), 122.24 and 119.67 (2C and 2C, C(1,9)H and C(3,6)H), 112.25 (2C, = C(11,14)H_2_), 37.07 (1C, C(9)H_2_), 22.22 (2C, C(12,15)H_3_) ppm.

**2,8‐di(prop‐1‐en‐2‐yl)‐dibenzothiophene (DIDBT)**. *M* 261 g mol^−1^. White solid, mp 94°C. Actual yield 0.734 g. Yield 95%.

ATR‐FTIR: ν = 3084 (m; vinyl = C─H stretch, and w shoulder extending to 3030; aromatic = C─H stretch), 2972, 2944, 2917 (m; aliphatic ─C─H stretch), 1774 (w; overtone of aromatic C═C stretch), 1622 (m; vinylic C═C stretch), 1546 (m; aromatic C═C stretch), 866, 810 (s; deformation of aromatic = C─H) cm^−1^.

^1^H NMR (400 MHz, CDCl_3_): δ = 8.22 (d, 2H, *J*_meta_ = 1.7 Hz, C(1,9)H), 7.78 (d, 2H, *J*_ortho_ = 8.4 Hz, C(4,6)H), 7.60 (dd, 2H, *J*_ortho_ = 8.4 Hz and *J*_meta_ = 1.7 Hz, C(3,7)H), 5.50 (app s, 2H, C(11,14)H trans to CH_3_), 5.18 (app t, 2H, *J*_long range_ = −1.4 Hz, C(11,14)H cis to CH_3_), 2.29 (app s, 6H, C(12,15)H_3_) ppm.

^13^C NMR (400 MHz, CDCl_3_): δ = 143.35 (2C, = C(10,13)‐CH_3_ quaternary), 139.05 (2C, C(2,8) quaternary), 138.08 (2C, C (9a,9b) quaternary, 135.76 (2C, C(4a,5a)‐S quaternary), 124.74 (2C, C(4,6)H), 122.61 and 118.46 (2C and 2C, C(1,9)H and C(3,7)H), 112.78 (2C, = C(11,14)H_2_), 22.37 (2C, C(12,15)H_3_) ppm.

### 4.3 Synthesis of Inverse Vulcanized Polymers (IVPs)

Random Sulfur‐rich co‐ and ter‐polymers were synthesized via inverse vulcanization (IV) of Sulfur (S) with various aromatic comonomers at different number of isopropenyl functionalities (see Chart 1 in the Introduction) and in different ratios among them, keeping the S content equal to 50 or 60 wt.%.

In a typical IV reaction, elemental Sulfur (ca. 2 g) is added to a Schlenk reaction vessel equipped with a magnetic stirring, and kept under a gentle Argon flow. The reaction vessel is placed in a heated oil bath at 160°C while stirring. Once the Sulfur has melted, the isopropenyl monomer(s) is added, and the temperature is raised to 180°C and maintained for 2 h. During heating, the reaction mixture (molten Sulfur acts both as solvent and reactant) of the inverse vulcanized polymer (IVP) being formed becomes rapidly highly viscous, preventing the stir bar from continuing to mix in about 10 min reaction time, see Figure S7. At the end of the 2‐h period, the temperature is lowered to 130°C and maintained at that level for another 24 h. After this curing time, the system is cooled to room temperature. Following this polymerization protocol, the IVP products are achieved in practically quantitative yield and used without further purification.

### 4.4 DBR Fabrication

DBR structures were fabricated by spin‐casting 11 alternate layers (that is 5.5 bilayers) on substrate at constant deposition volume (100 µL) and rotation speed (150 rps). As high‐*n* component, a solution (40 mg/mL total concentration) obtained dissolving in toluene the IVP sample (20 mg mL^−1^) and polystyrene (PS, 20 mg mL^−1^, *n* at 600 nm 1.57) was used; while as low‐*n* counterpart cellulose acetate (CA, 25 mg mL^−1^, *n* at 600 nm 1.46) dissolved in 4‐hydroxy‐4‐methylpentan‐2‐one was employed. We notice that the real IVPs solutions contain an undissolved precipitate. Solutions used for spectroscopy and DBR preparation are then the supernatant of such real mixture, and their real concentration is unknown. Moreover, the amount of IVPs in the supernatant is higher for fresh prepared IVPs solutions and decreases over time as highlighted in the main text. The spectral features and fluorescence properties of the supernatant are however very clear and stable over time.

### 4.5 Metamaterials Patterning

Replica molding microstructures were prepared by heating selected IVP samples above their *T*_g_ and imprinting them on a glass diffraction grating of period of 4 µm or on a plastic Fresnel lens of period 180 µm. Optical profilometry images were obtained by Sensofar S Neox profilometer. The Back Focal Plane (BFP) imaging system is described in details in Ref. [65].

### 4.6 Characterization Methods

Fourier transform infrared spectroscopy in attenuated total reflectance mode (ATR‐FTIR) was performed on both comonomers and IVPs operating with a PerkinElmer Spectrum Two spectrometer, recording transmittance spectra in the wavenumber range 4000–400 cm^−1^ (16 scans, resolution 0.5 cm^−1^).

^1^H NMR spectra of synthesized comonomers were acquired at room temperature in CDCl_3_ (internal chemical shift reference 0.03% TMS) with a JEOL ECZ400R/S3 400 MHz spectrometer on a 10 mg sample using a 5 mm probe.

^13^C NMR spectra were performed on the same 400 MHz instrument at room temperature in CDCl_3_ (also used as internal chemical shift reference), operating in single pulse decoupled gated NOE mode on a 20 mg sample using a 5 mm probe.

Dynamic thermogravimetric analysis (TGA) measurements on IVPs was carried out with a PerkinElmer TGA8000 instrument. Specimens (two or three replicas for each composition) of about 15 mg were heated in the temperature range from 40 up to 700°C at 10°C min^−1^ under N_2_ and from 700°C to 850°C at 20°C min^−1^ under O_2_ (gas flow 35 mL min^−1^).

Differential scanning calorimetry (DSC) measurements on IVP samples (for each composition, two measurements were taken on specimens of ca. 10 mg) were performed either with a Mettler DSC 821^e^ or a Mettler DSC3+ calorimeter under N_2_ atmosphere (flow 40 mL min^−1^) imposing a first heating‐controlled cooling‐second heating thermal cycle at scan rate of 20°C min^−1^: minimum (from −30°C to 30°C) and maximum (from 160°C to180°C) temperatures explored were adjusted depending on the reasonably expected *T*_g_ values.

Absorbance and fluorescence spectra were collected on IVP solutions of ca. 0.2 mg mL^−1^ using an in‐house‐assembled optical set‐up. For absorbance, a Deuterium–Halogen light source (DH 2000 BAL) and an Avantes Avaspec 2048‐EVO spectrometer (250–1050 nm with resolution 1.4 nm) connected through optical fibers were used. Fluorescence response was collected with the same detector exciting the systems with a blue laser source (Changchun New Industries Optoelectronic Tech, emission at 448 nm, 40 mW power). The fluorescence signal was filtered with a 450 nm long‐pass filter (Semrock) to eliminate the excitation source from the measured spectra.

## Funding

PRIN 2020 Project “PETALS Polymer mETamateriALs for nanophotonicS” (prot. 2020TS9LXS) funded by the Italian Ministry for Universities and Research (MUR).

## Conflicts of Interest

The authors declare no conflicts of interest.

## Supporting information




**Supporting File**: marc70230‐sup‐0001‐SupMat.docx.

## Data Availability

The data that support the findings of this study are available in the supplementary material of this article.
