# Supplementary material for: Novel Sulfur‐Rich Polymers from Inverse Vulcanization as Functional Building Blocks for Photonics
Source: Macromol Rapid Commun. 2026 Feb 8;47(13):e00957. doi: 10.1002/marc.202500957 (PMC13331540; doi:10.1002/marc.202500957)
Supplement: Supplementary file 1 — Supporting File: marc70230‐sup‐0001‐SupMat.docx. [file MARC-47-e00957-s001.docx]

Supporting Information

Novel Sulfur-rich Polymers from Inverse Vulcanization as Functional Building Blocks for Photonics

Raimondo Insogna, Fabiano Martorelli, Daniela Di Fonzo, Martina Martusciello, Roberto Utzeri, Angelo Angelini, Davide Comoretto, and Paola Stagnaro*


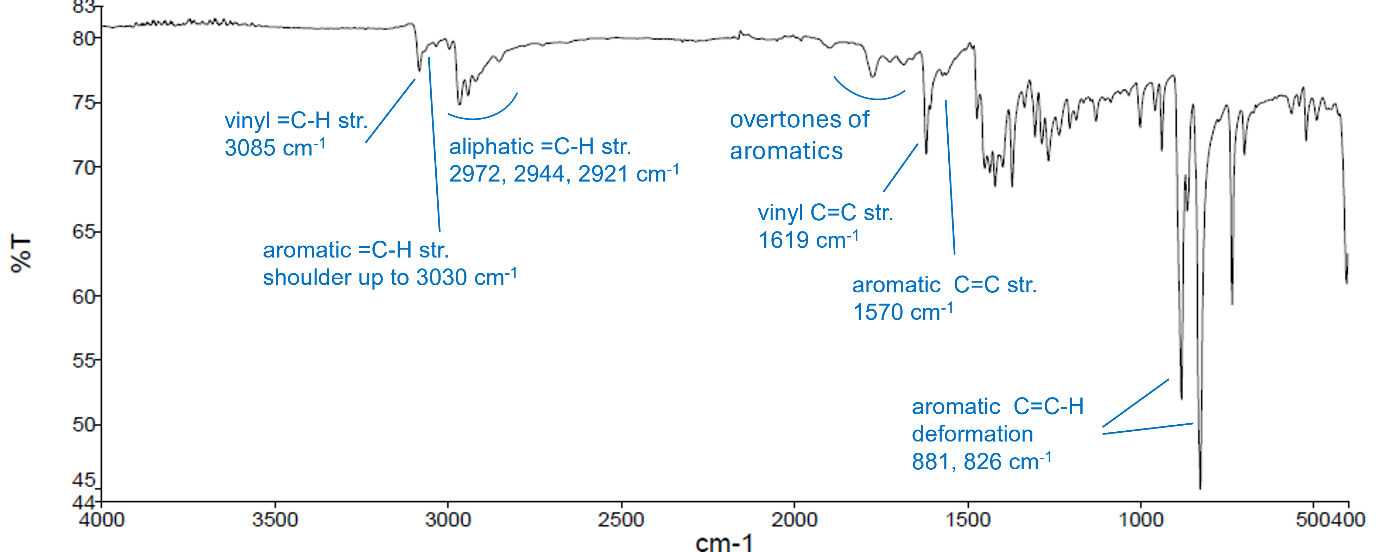


**Figure S1.** Infrared transmittance spectrum of 2,7-diisopropenylfluorene (**DIF**) acquired in ATR-FTIR mode.


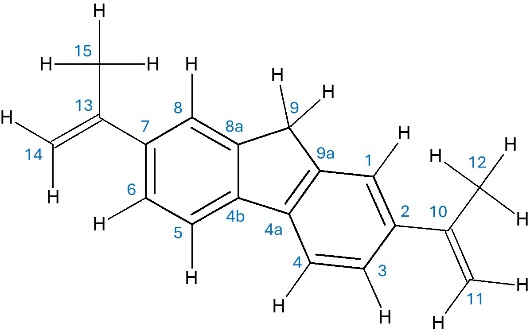

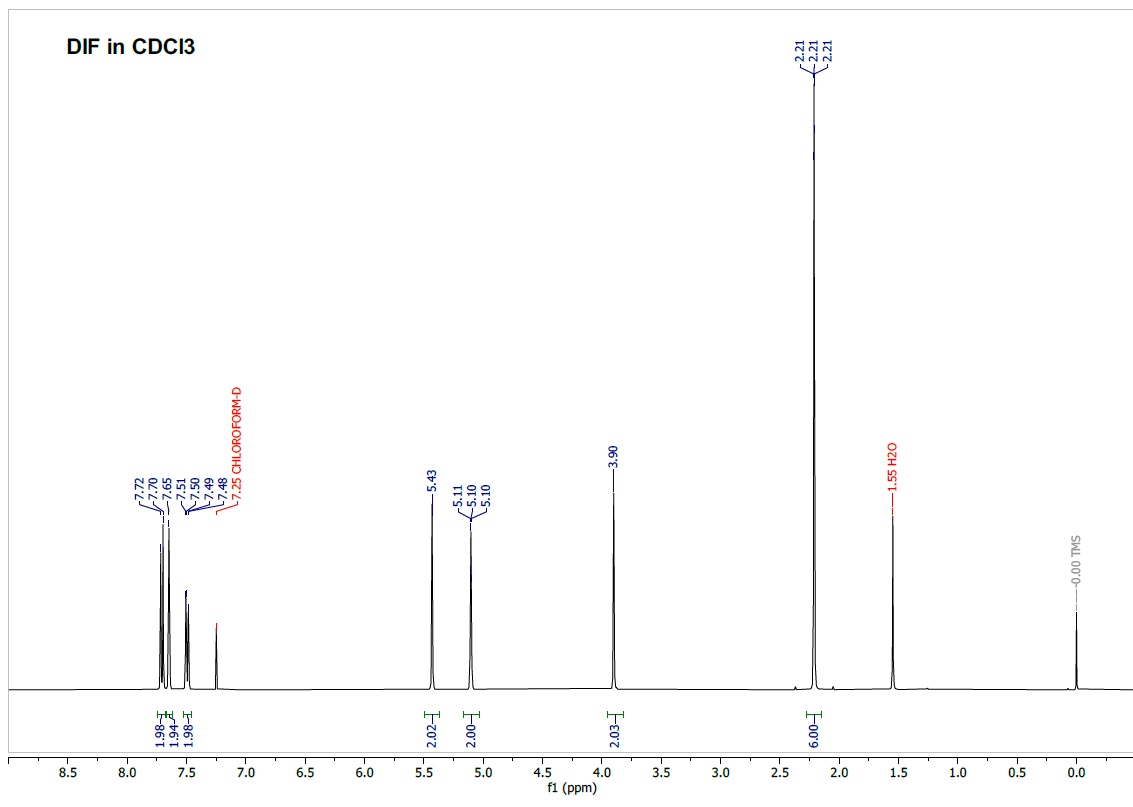


**Figure S2a.** ^1^H NMR spectrum of 2,7-diisopropenylfluorene (**DIF**) in CDCl_3_, with signals assignments (structure numbering in the figure inset).


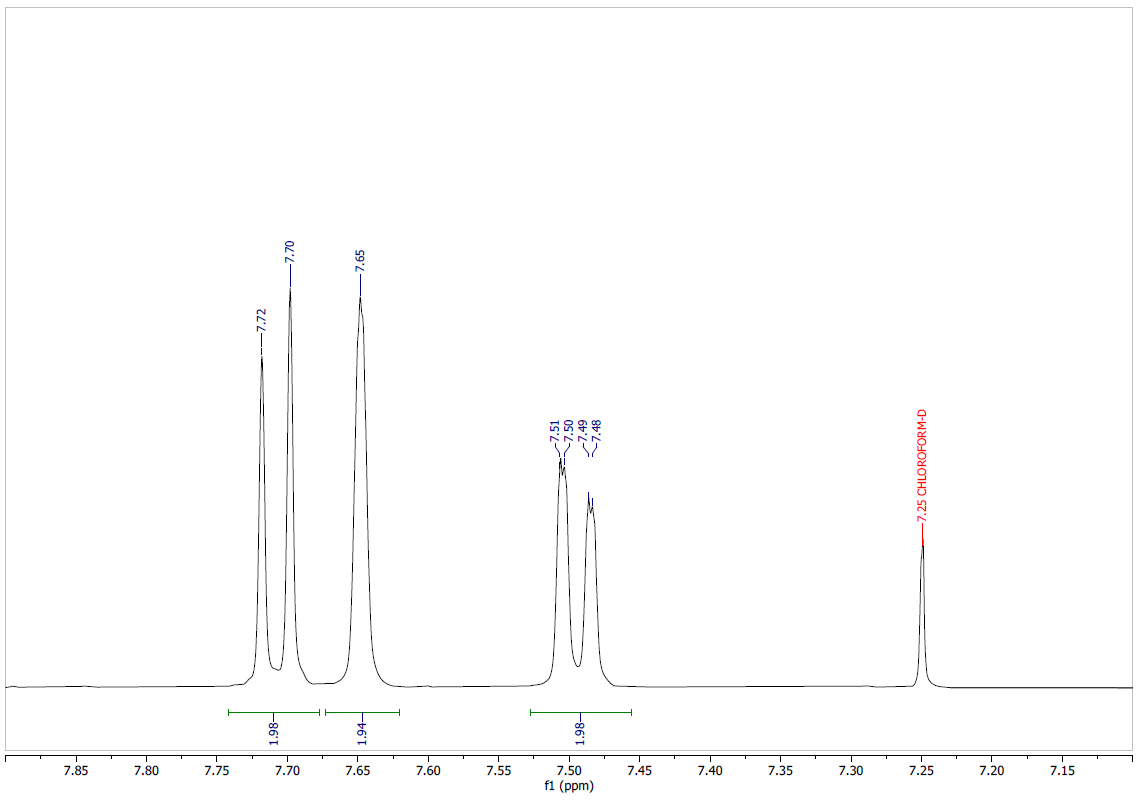


**Figure S2b.** ^1^H NMR spectrum of 2,7-diisopropenylfluorene (**DIF**) in CDCl_3_ (aromatic H zone).


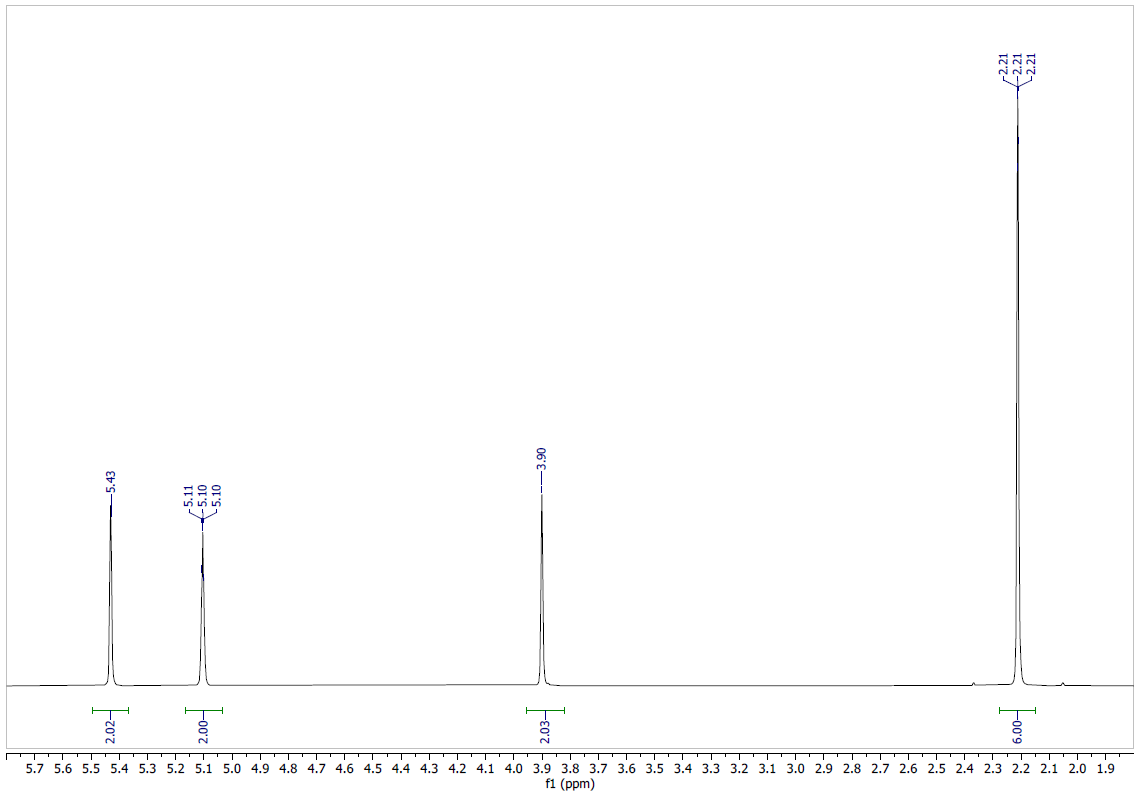


**Figure S2c.** ^1^H NMR spectrum of 2,7-diisopropenylfluorene (**DIF**) in CDCl_3_ (vinylic and aliphatic H zone).


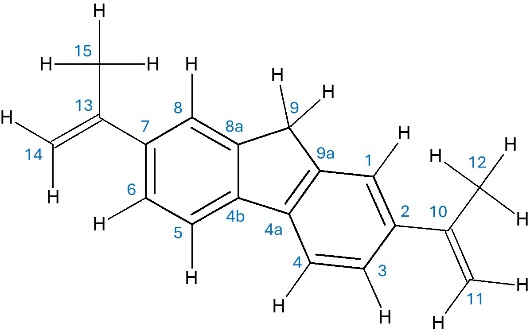


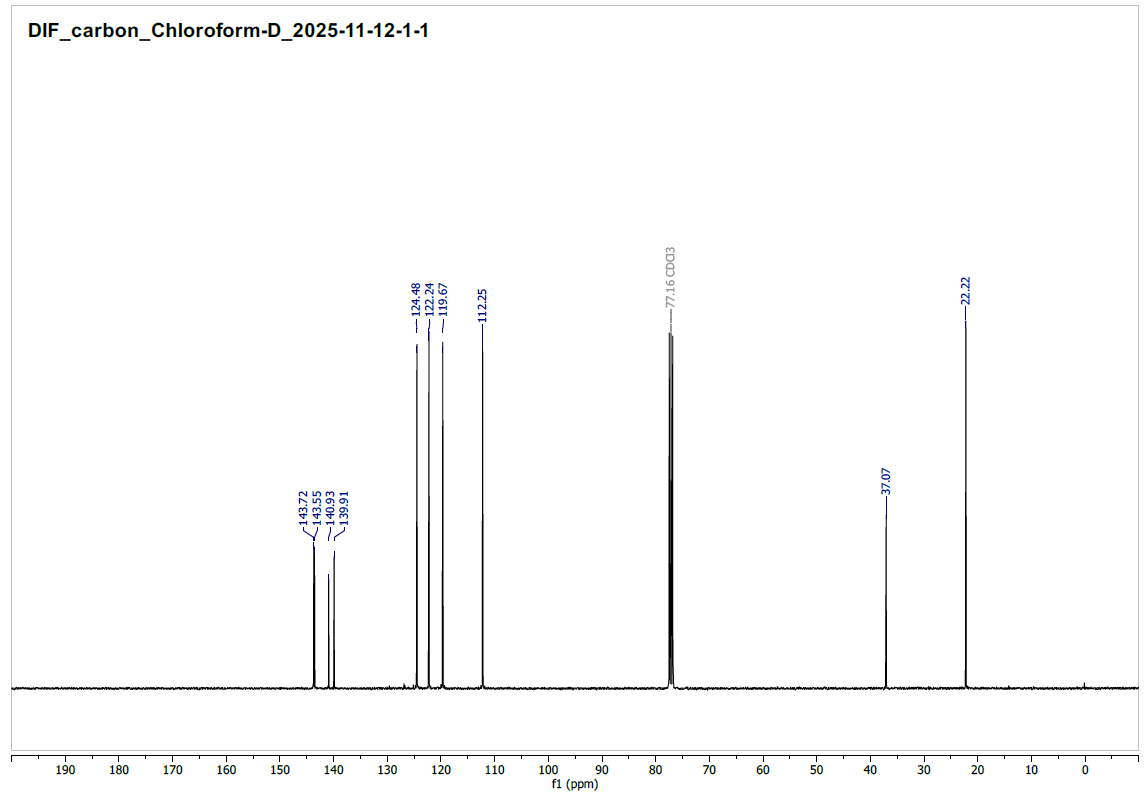


**Figure S3a.** ^13^C NMR spectrum of 2,7-diisopropenylfluorene (**DIF**) in CDCl_3_, with signals assignments (C numbering in the figure inset).


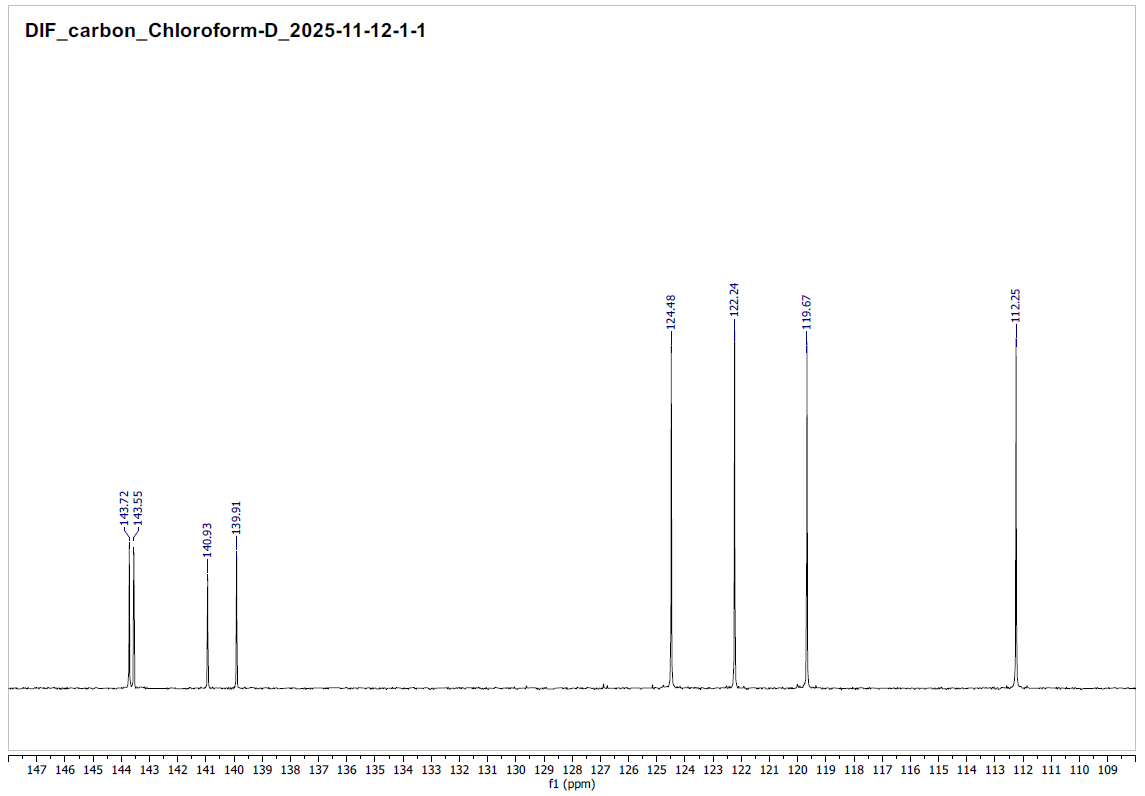


**Figure S3b.** ^13^C NMR spectrum of 2,7-diisopropenylfluorene (**DIF**) in CDCl_3_ (unsaturated C zone).


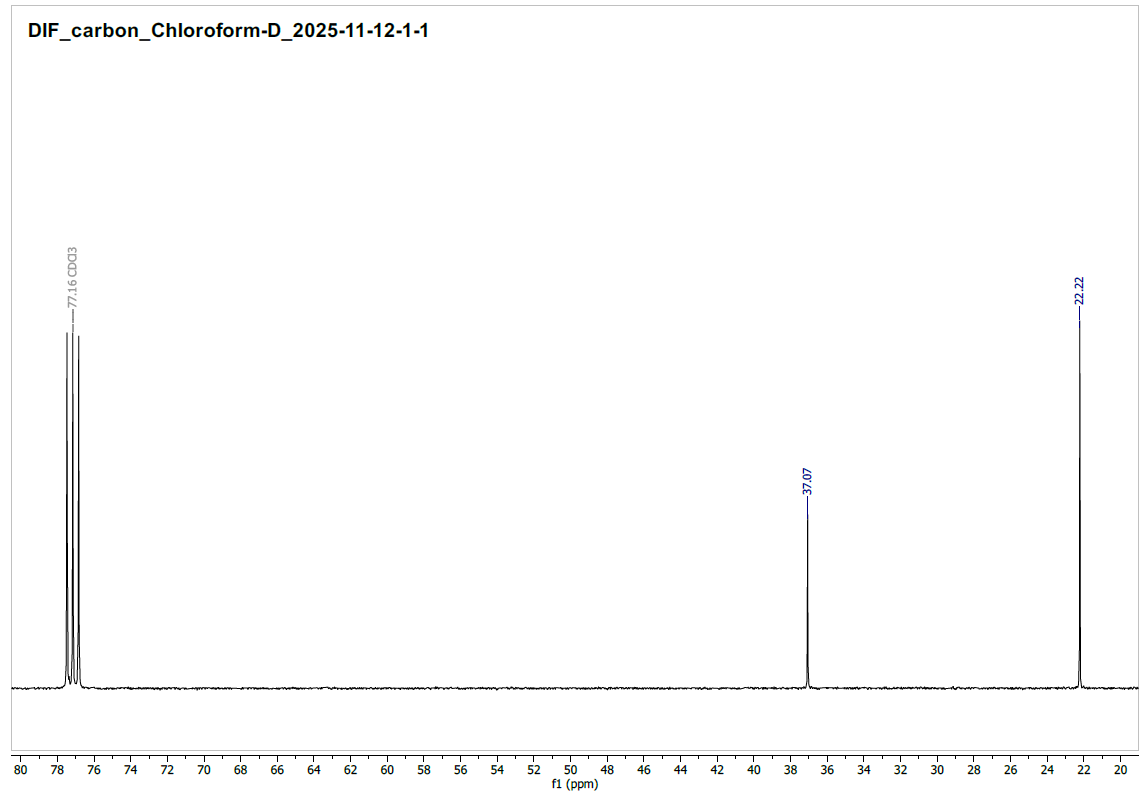


**Figure S3c.** ^13^C NMR spectrum of 2,7-diisopropenylfluorene (**DIF**) in CDCl_3_ (aliphatic C zone).


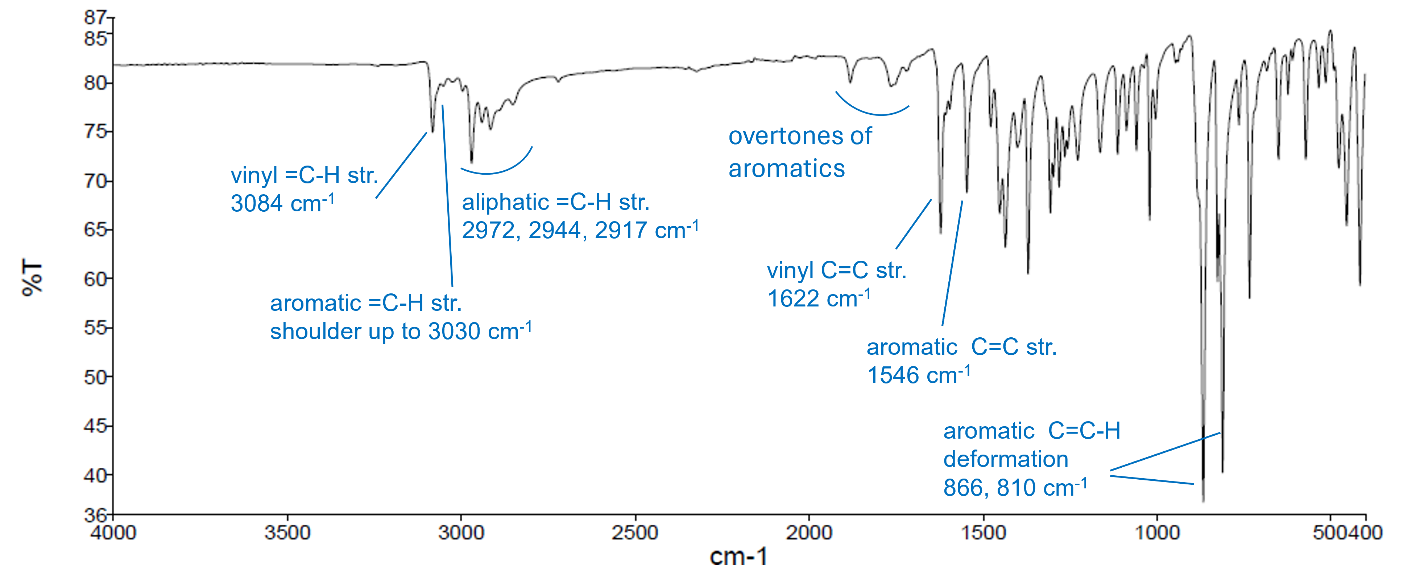


**Figure S4.** Infrared transmittance spectrum of 2,8-diisopropenyldibenzothiophene (**DIDBT**) acquired in ATR-FTIR mode.


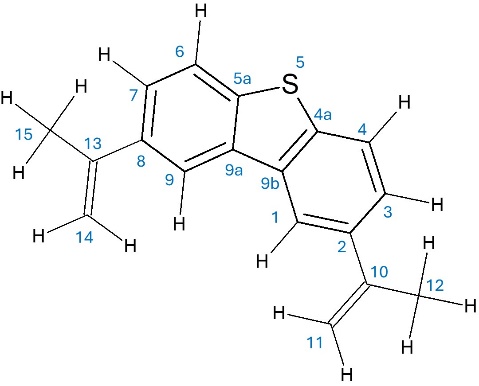

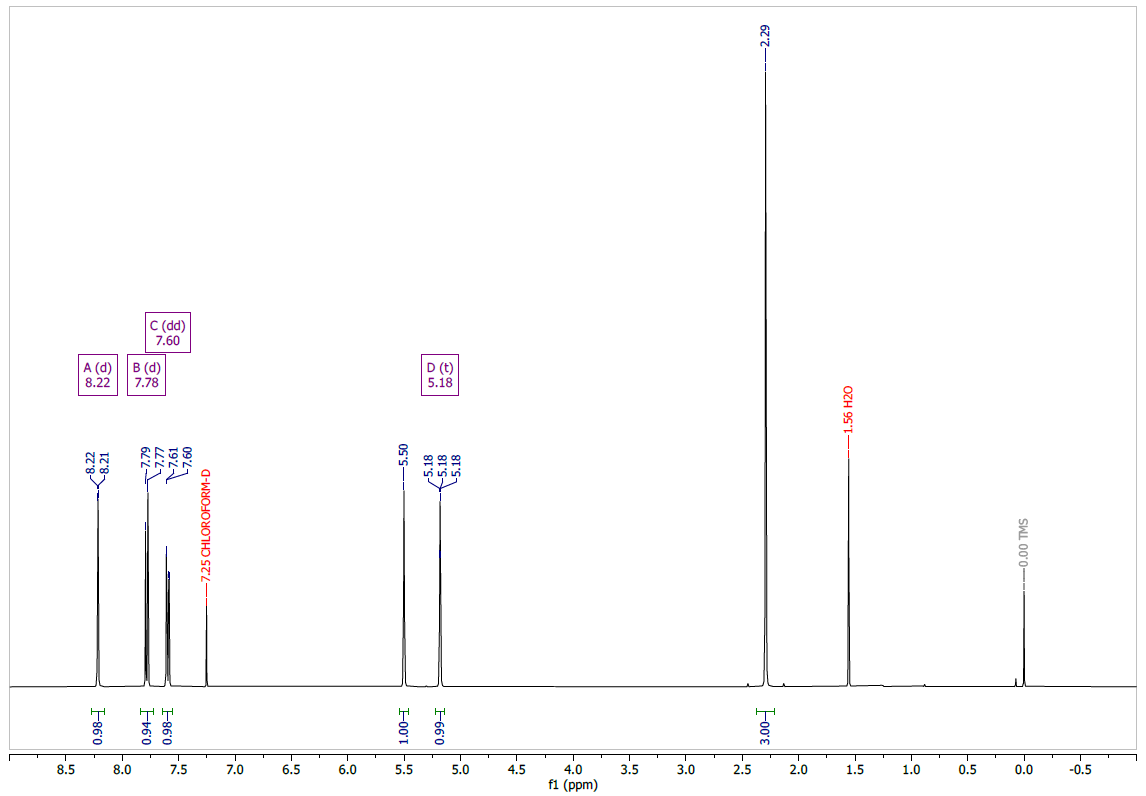


**Figure S5a.** ^1^H NMR spectrum of 2,8-diisopropenyldibenzothiophene (**DIDBT**) in CDCl_3_, with signals assignments (structure numbering in the figure inset).

**Figure S5b.** ^1^H NMR spectrum of 2,8-diisopropenyldibenzothiophene (**DIDBT**) in CDCl_3_, (aromatic H zone).


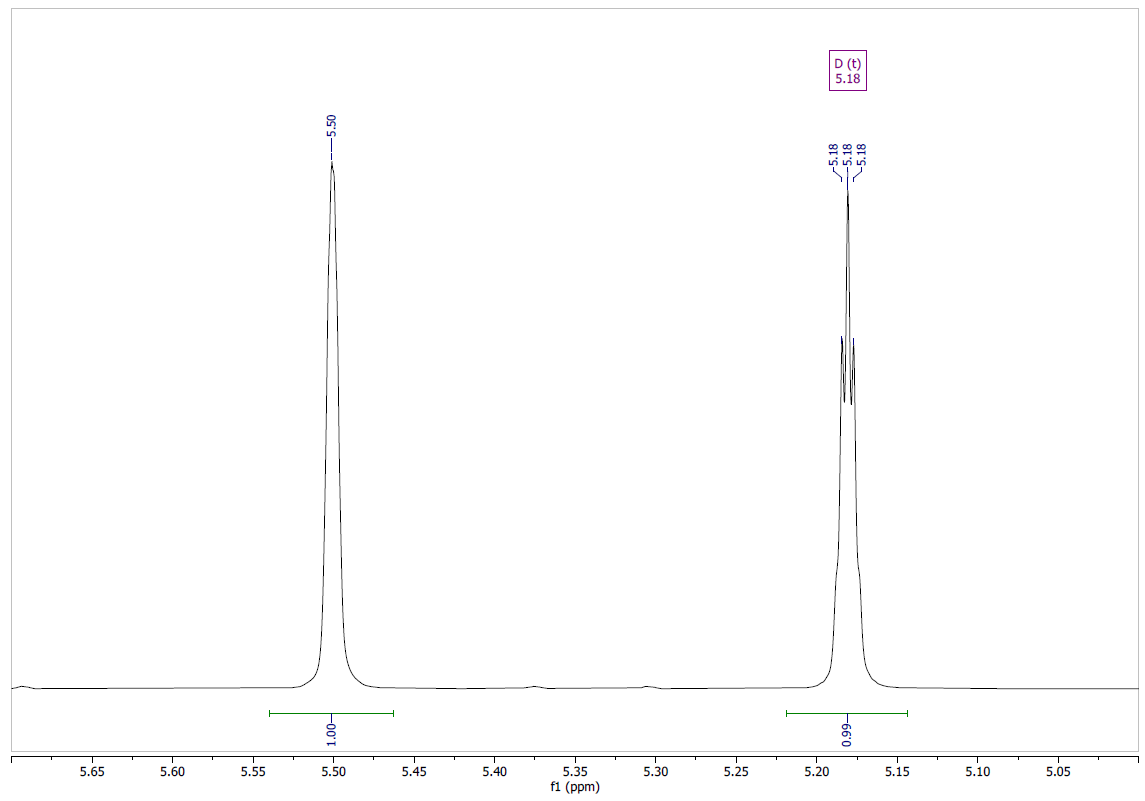


**Figure S5c.** ^1^H NMR spectrum of 2,8-diisopropenyldibenzothiophene (**DIDBT**) in CDCl_3_ (vinylic H zone).


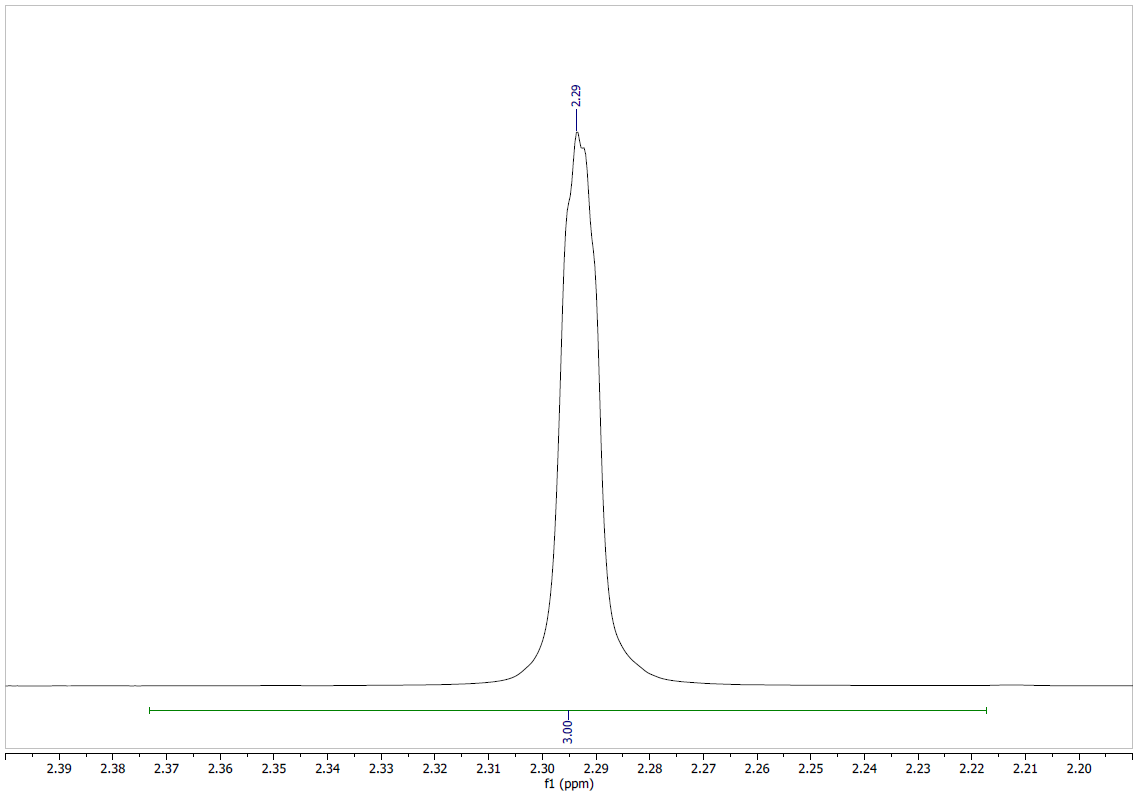


**Figure S5d.** ^1^H NMR spectrum of 2,8-diisopropenyldibenzothiophene (**DIDBT**) in CDCl_3_ (aliphatic H zone).


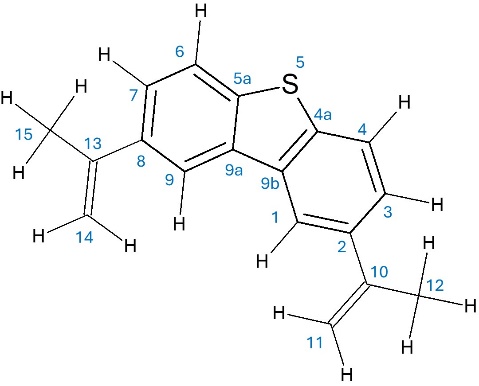


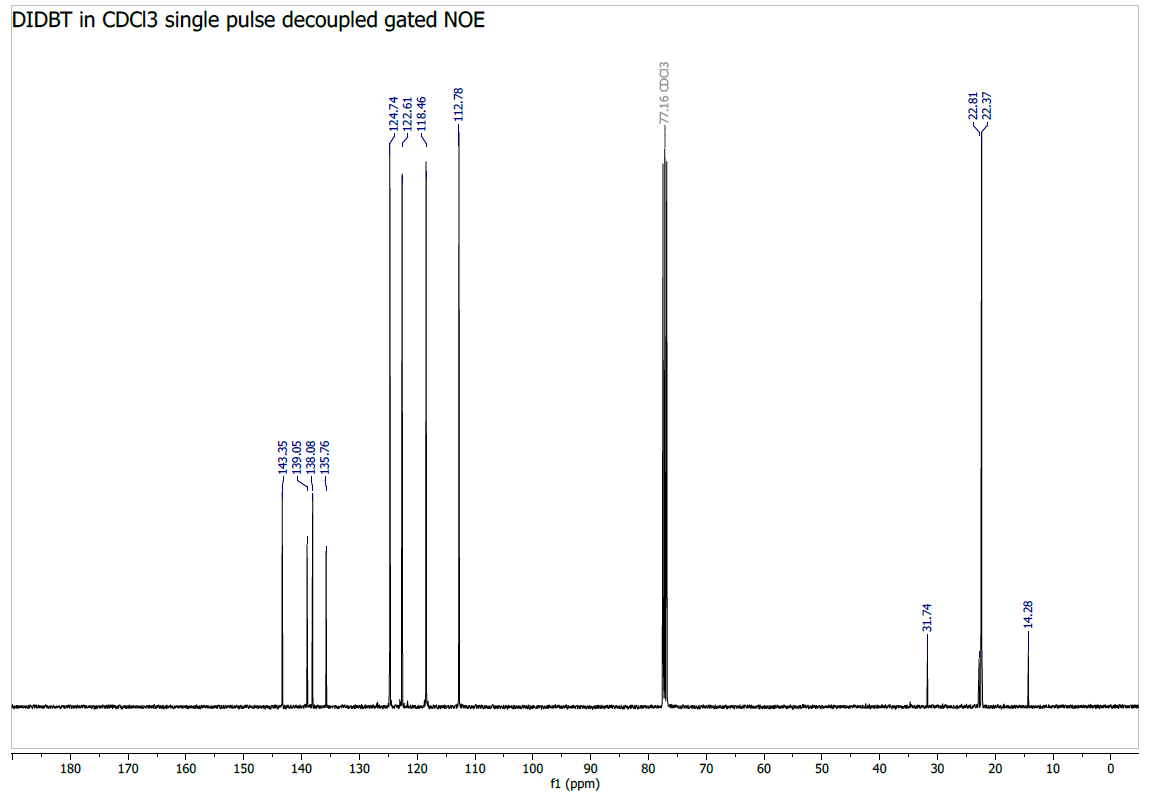


X

X

X

**Figure S6a.** ^13^C NMR spectrum of 2,8-diisopropenyldibenzothiophene (**DIDBT**) in CDCl_3_, with signals assignments (C numbering in the figure inset).


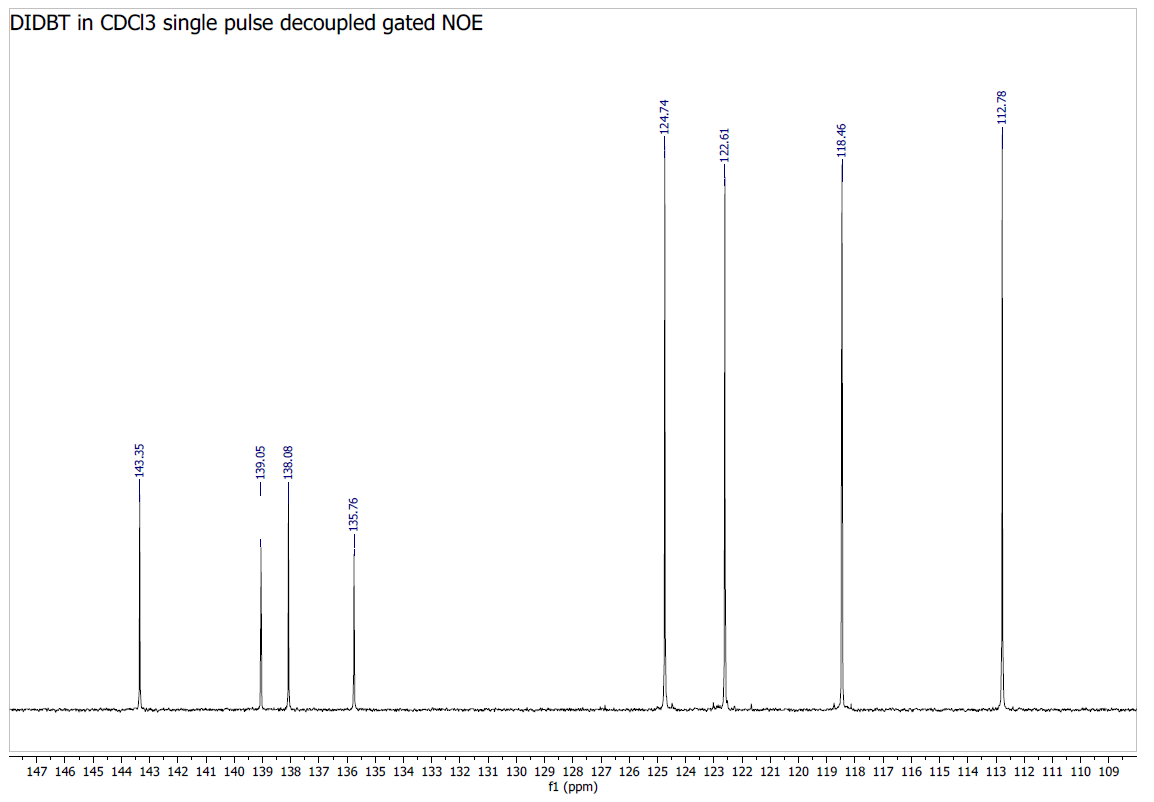


**Figure S6b.** ^13^C NMR spectrum of 2,8-diisopropenyldibenzothiophene (**DIDBT**) in CDCl_3_ (unsaturated C zone).


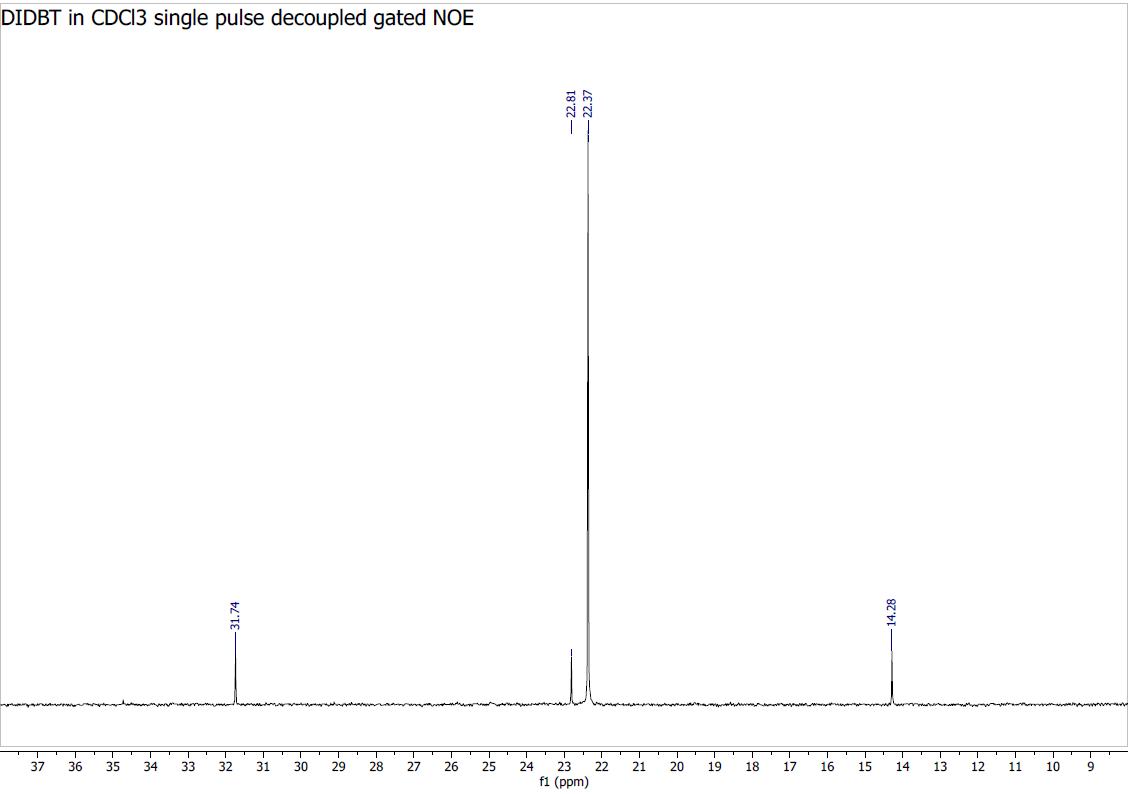


X

X

X

**Figure S6c.** ^13^C NMR spectrum of 2,8-diisopropenyldibenzothiophene (**DIDBT**) in CDCl_3_ (aliphatic C zone).

**Figure S7.** Pictures of the reaction mixture taken throughout the IV course and an example of the final glassy IVP product achieved. Pictures are related to **S-TIB** 50/50 wt/wt system.


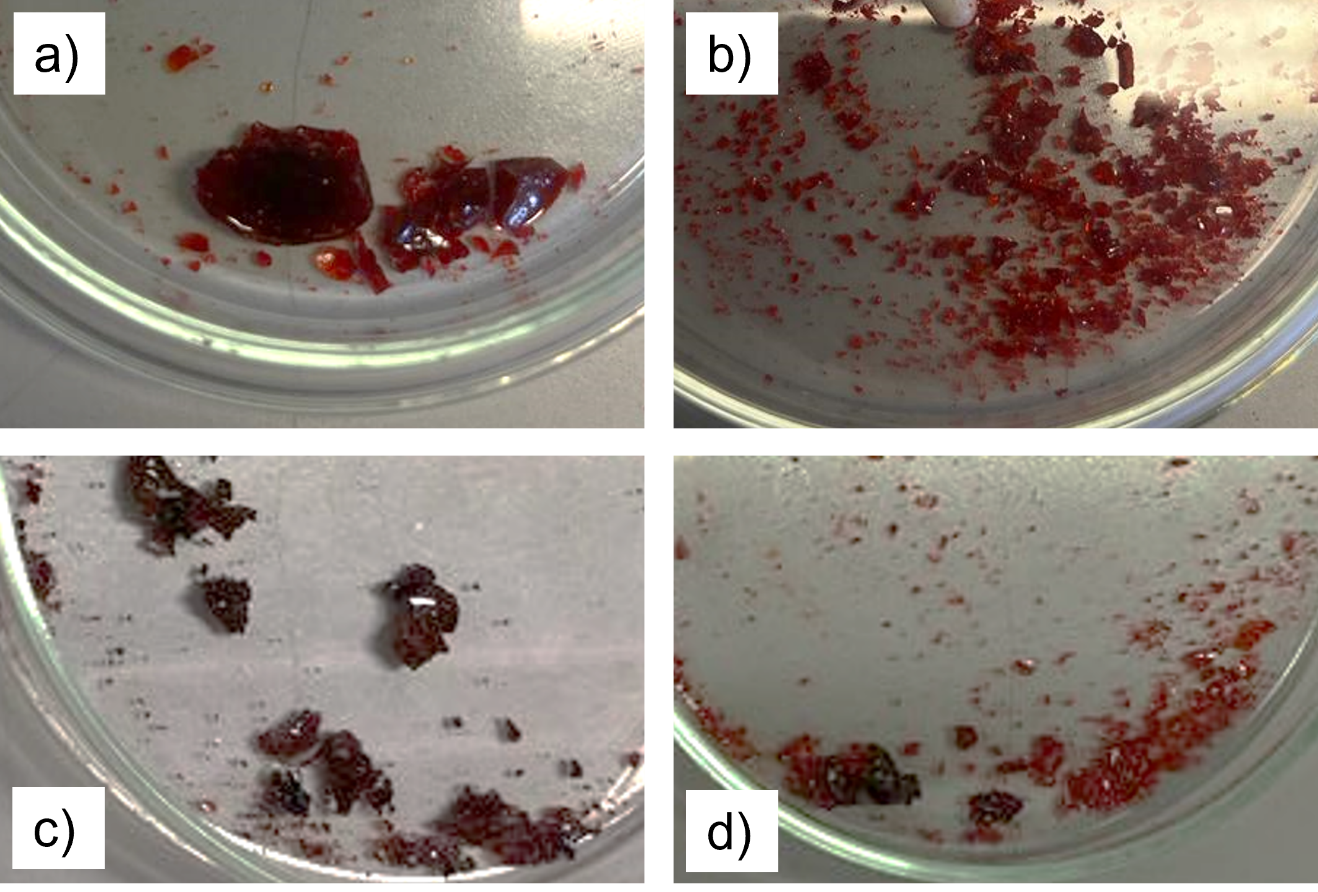


**Figure S8.** Pictures of some IVPs taken after different times from their synthesis at ambient conditions: (a) **S-TIB** 50/50 wt/wt after 1 month; (b) **S-TIB-αMS** 50/30/20 wt/wt after 2 weeks; (c) **S-DIF** 50/50 wt/wt after 4 months; (d) **S-DIDBT** 60/40 wt/wt after 3 months.

a)


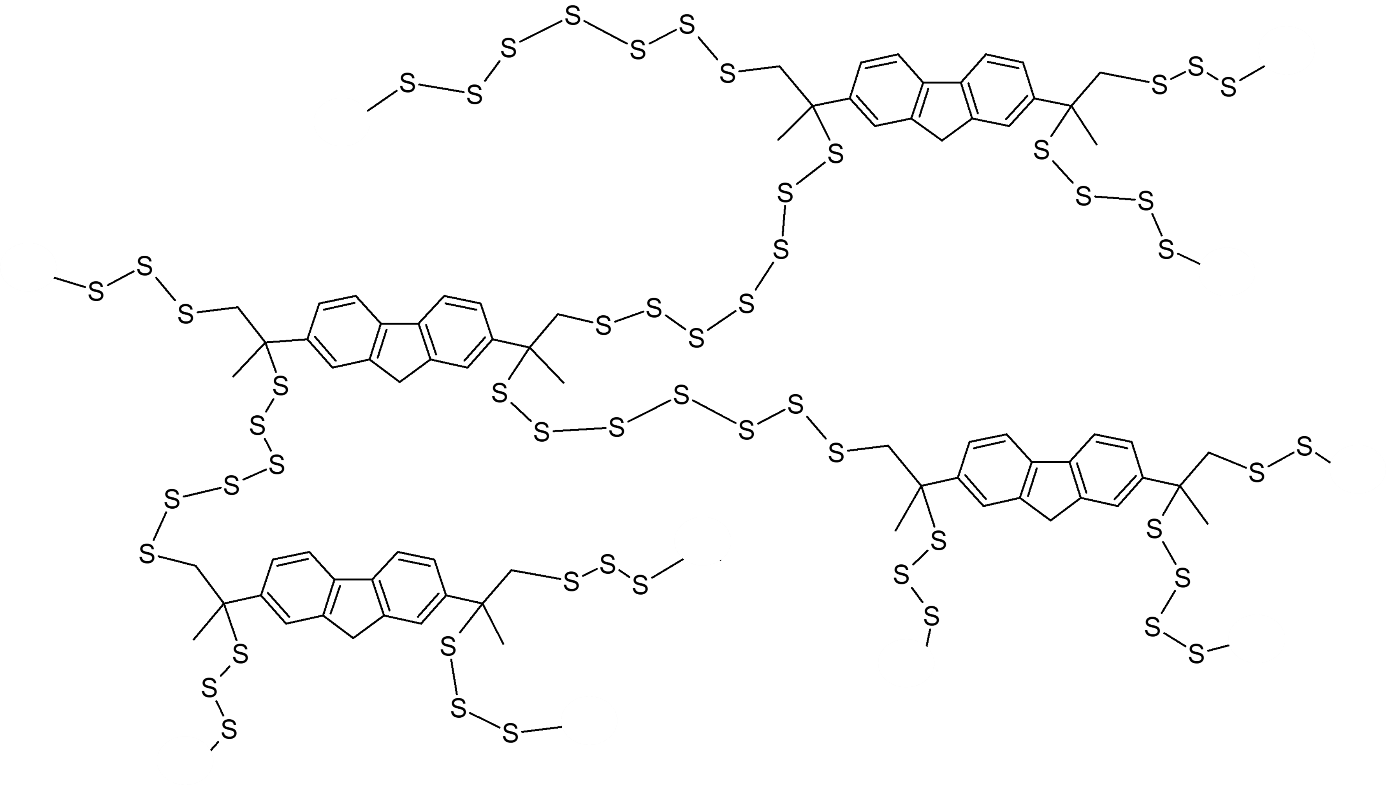


b)


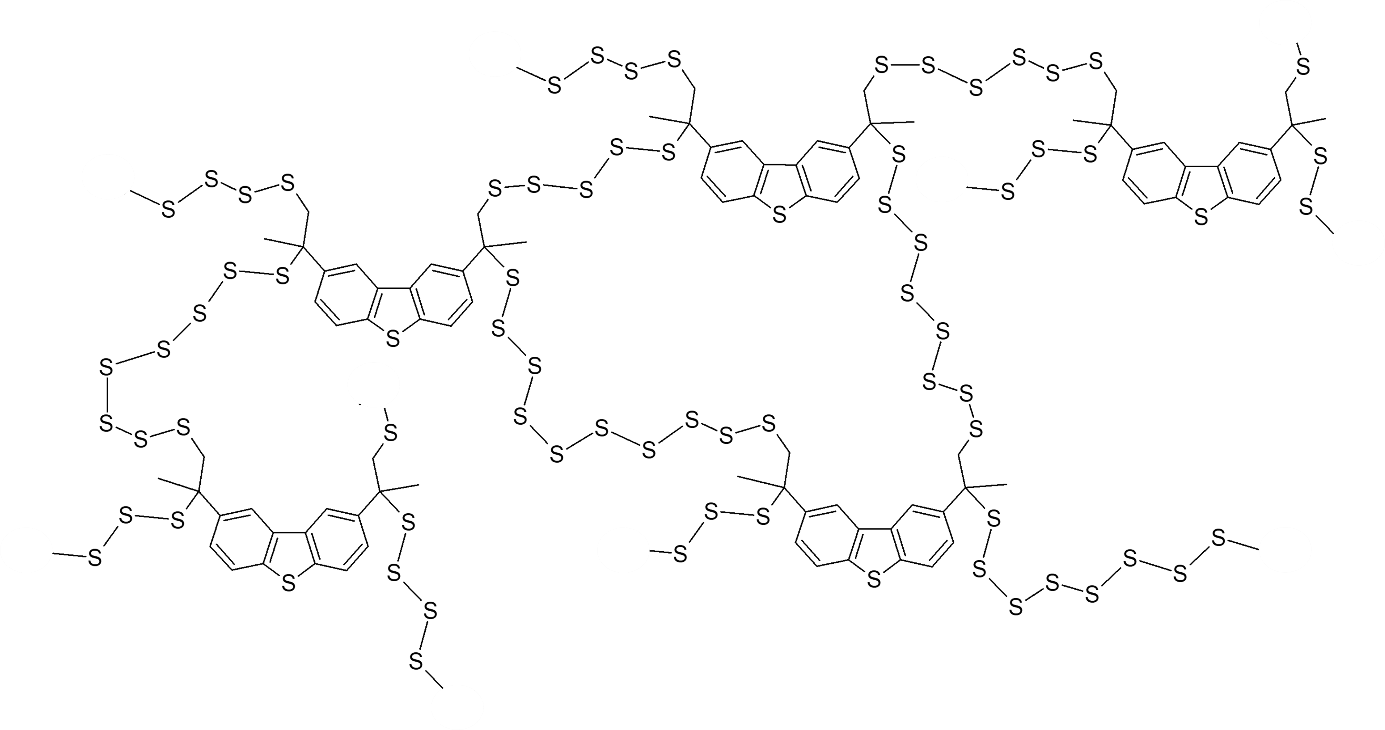


**Figure S9.** Random chemical structures of: (a) **S-DIF** and (b) **S-DIDBT** IVP systems.

a)


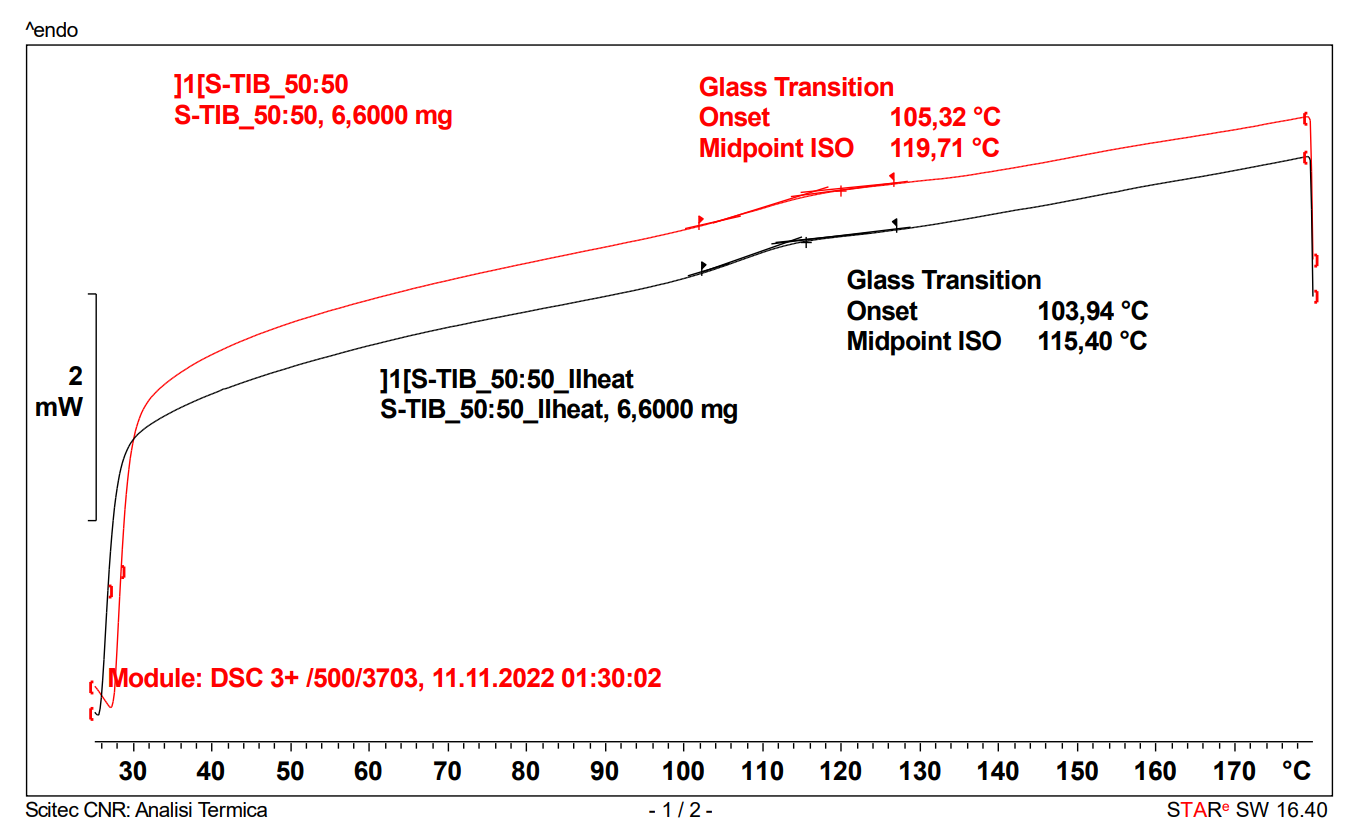


b)


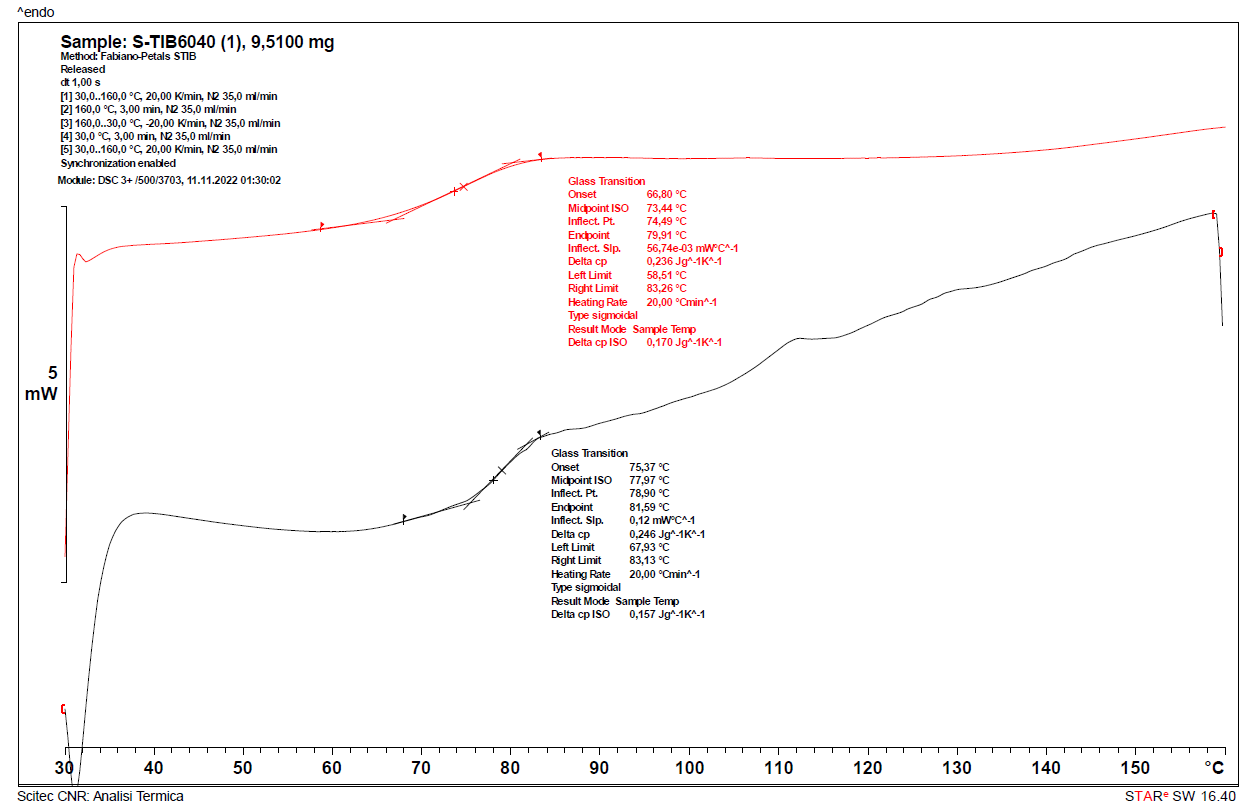


**Figure S10.** DSC first and second heating curves of: (a) **S-TIB** 50/50, and (b) **S-TIB** 60/40 wt/wt IVPs.

a)

^˄^endo


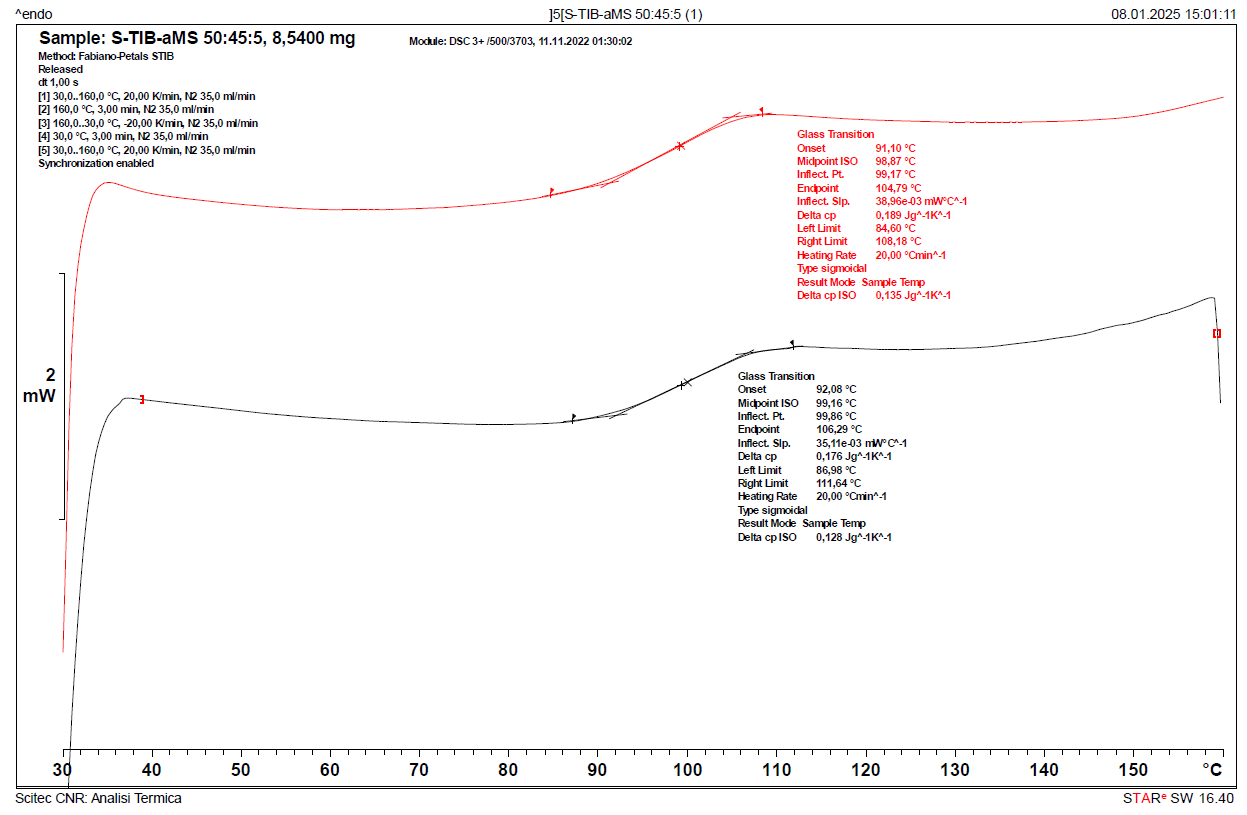


b)


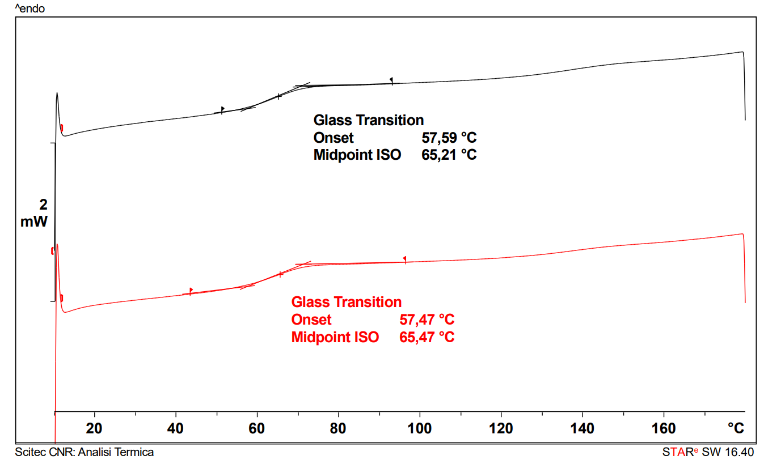


c)


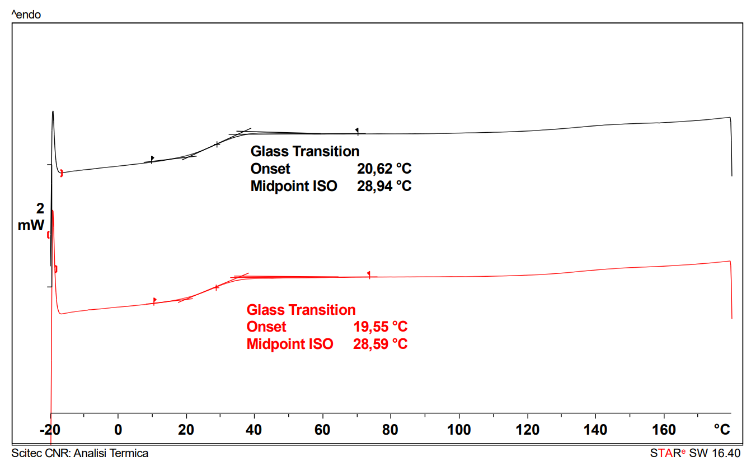


d)


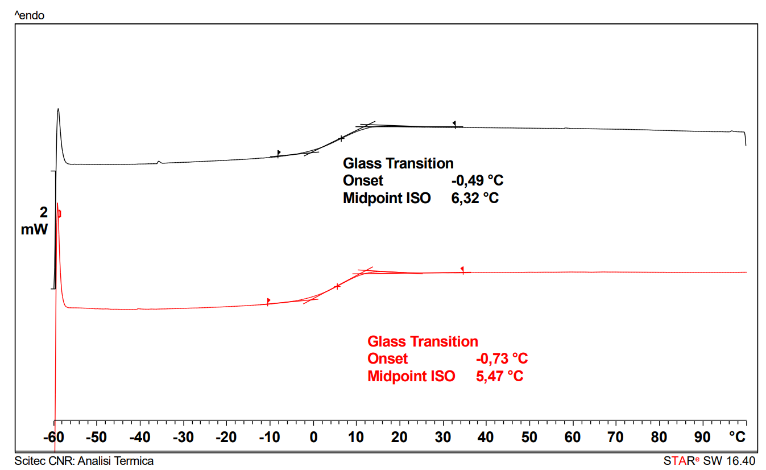


**Figure S11.** DSC first and second heating curves of: (a) **S-TIB-αMS** 50/45/5, (b) **S-TIB-αMS** 50/40/10, (c) **S-TIB-αMS** 50/30/20, and (d) **S-TIB-αMS** 50/20/30 wt/wt/wt IVPs.

c

a)


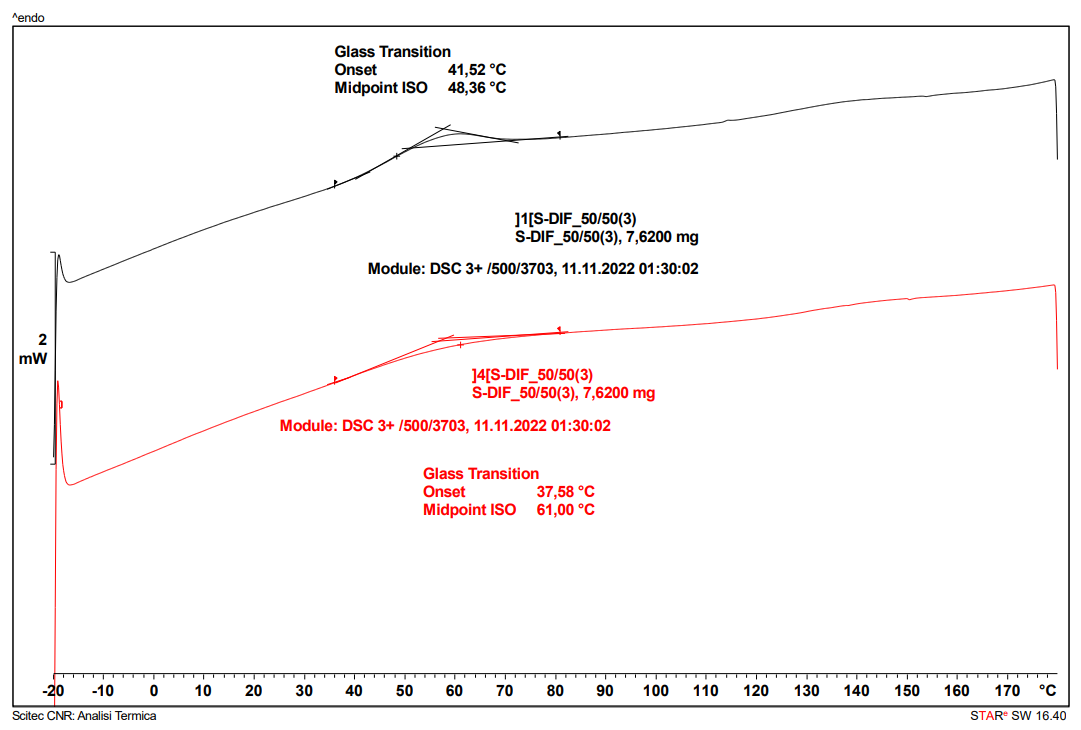


b)


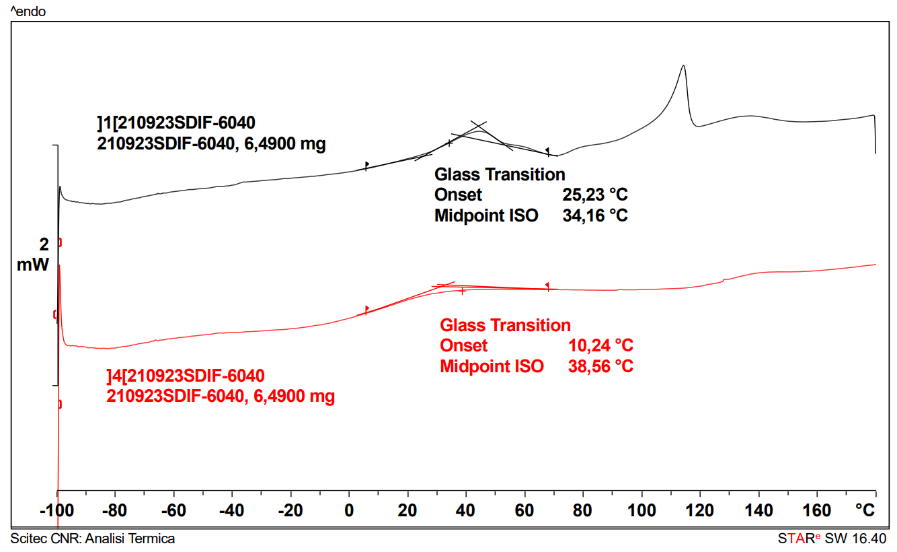


**Figure S12.** DSC first and second heating curves of: (a) **S-DIF** 50/50, and (b) **S-DIF** 60/40 wt/wt IVPs.

a)


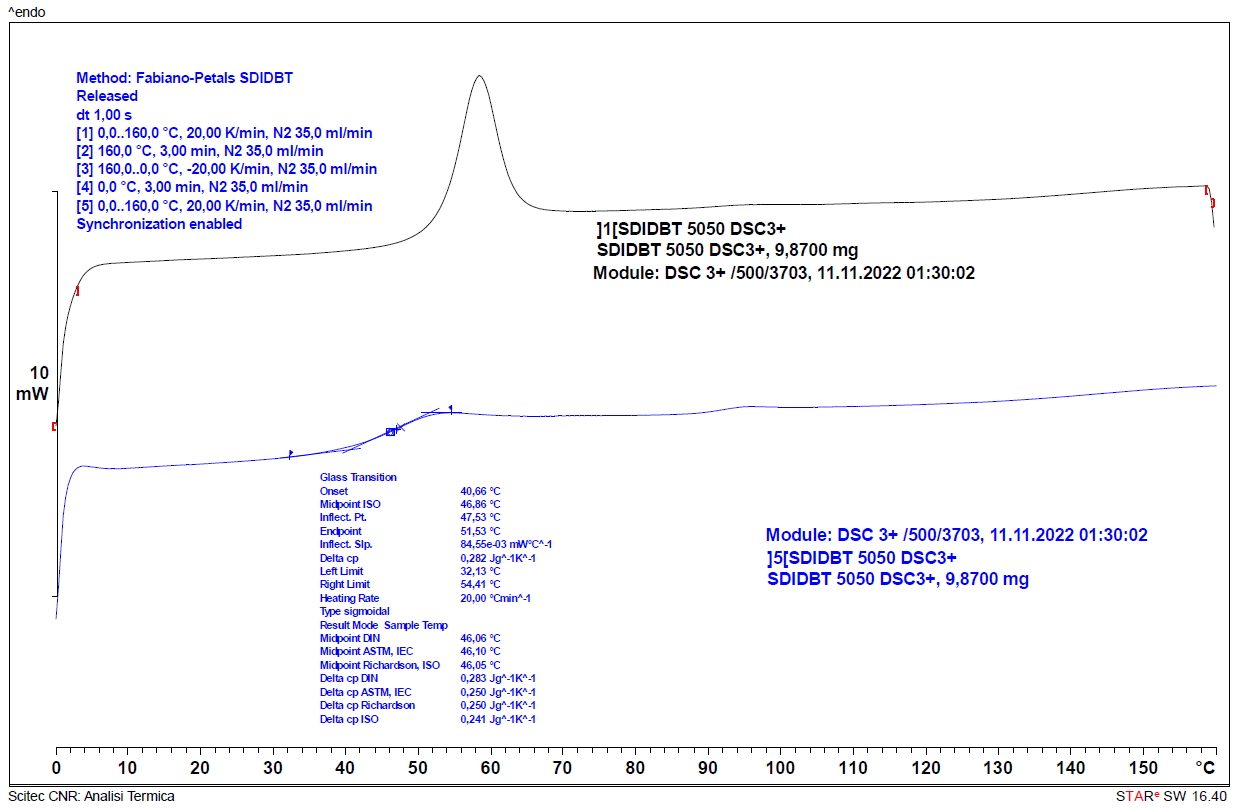


b)


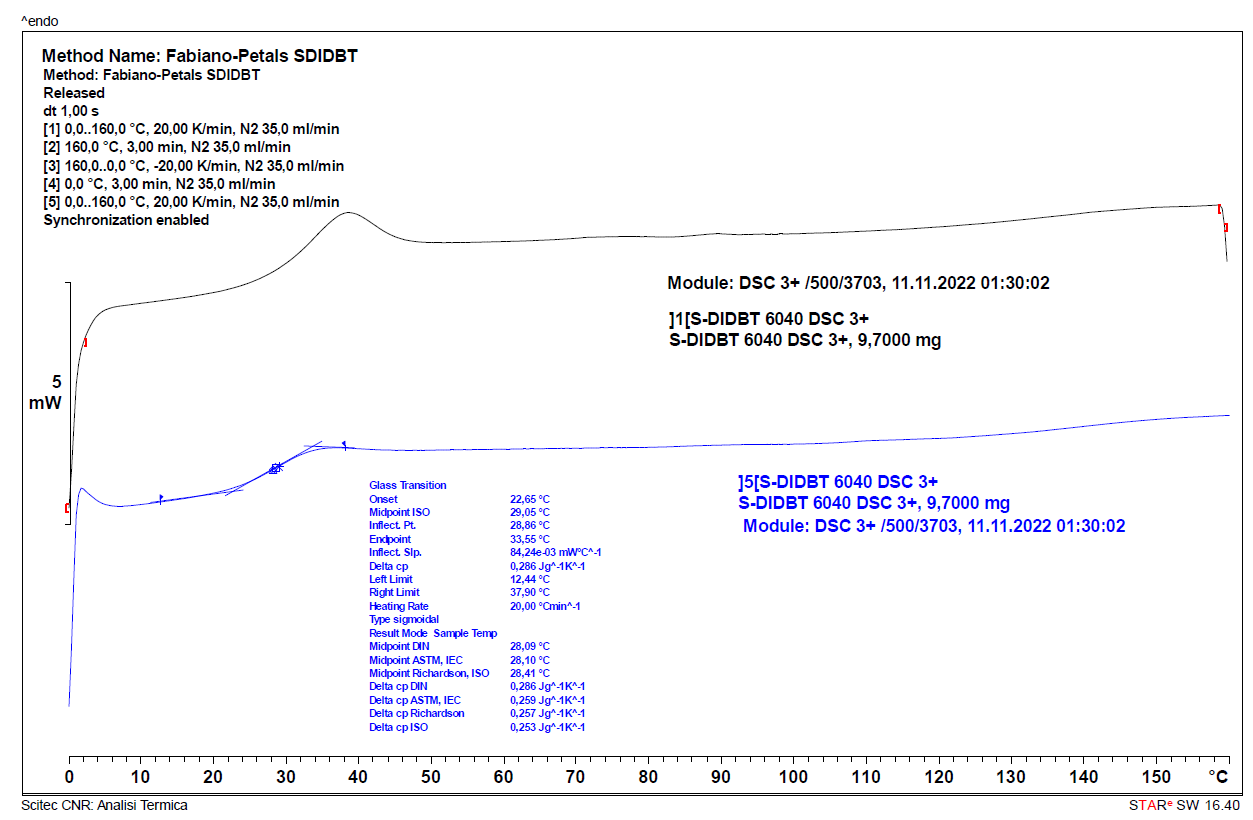


**Figure S13.** DSC first and second heating curves of: (a) **S-DIDBT** 50/50, and (b) **S-DIDBT** 60/40 wt/wt IVPs.

a)


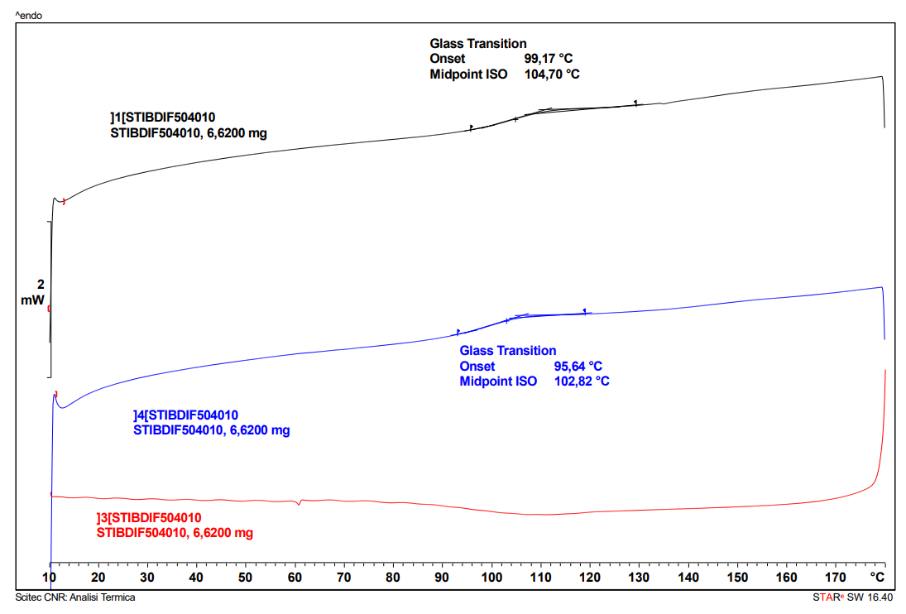


b)


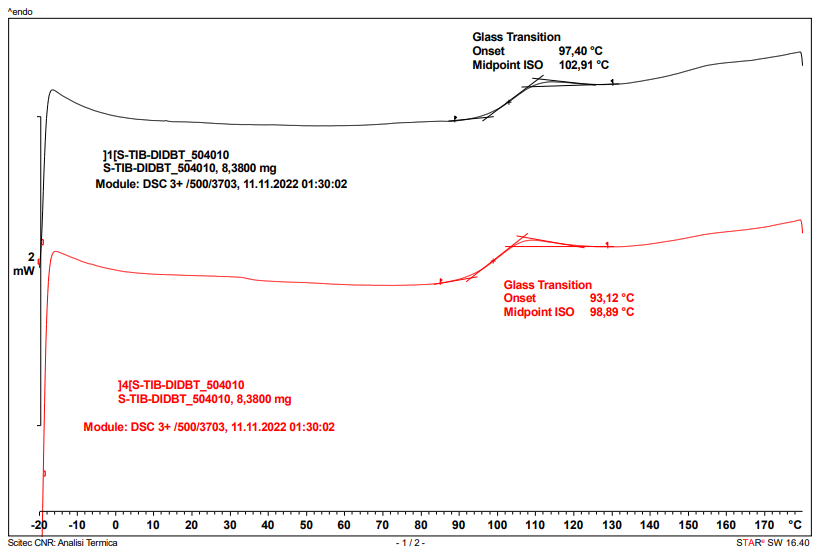


**Figure S14.** DSC first and second heating curves of: (a) **S-TIB-DIF** 50/40/10, and (b) **S-TIB-DIDBT** 50/40/10 wt/wt/wt IVPs.


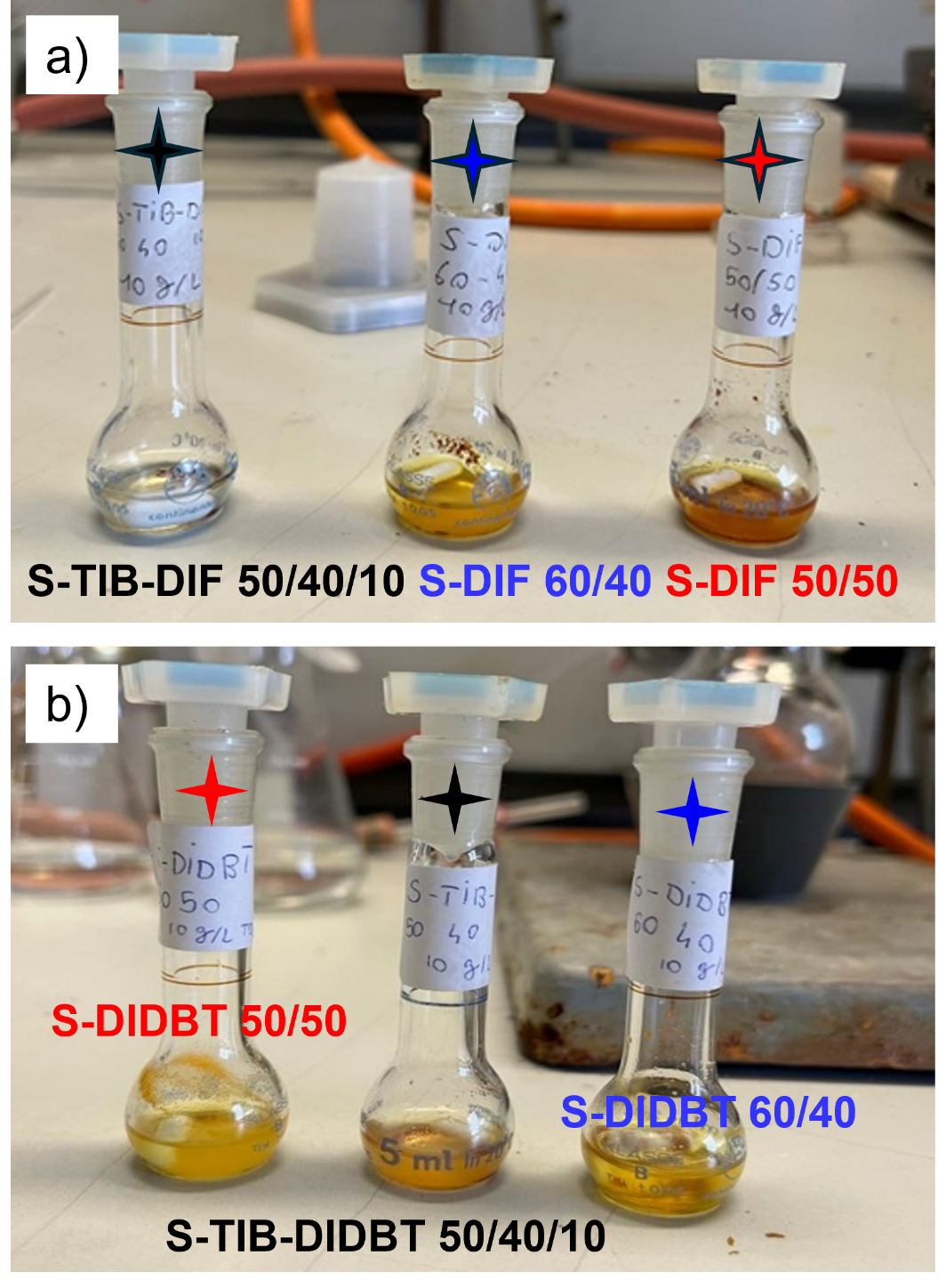


**Figure S15.** Solubility tests in toluene (10 mg mL^-1^ at 60 °C) on IVPs: (a) **S-DIF** series and (b) **S-DIDBT** series.

**Figure S16.** PL spectra of IVPs solutions recorded immediately after their synthesis.

**Comment on the current concerns on the determination of the complex refractive index**

According to our experience and know-how in the determination of the complex optical function of polymers and organic solids, a multiple techniques approach has to be used, by simultaneously using ellipsometry, reflectance and transmittance at different incident angles and for different polarizations. In particular, we noticed sometime ellipsometric determination alone might retain up to 10 % uncertainty.^[S1,S2]^ To overcome this problem, usually, ellipsometric determinations are combined with reflectance and transmittance spectra to deeply extend the spectral range in the UV and NIR. In particular, UV data allow to better fix film thickness, while NIR ones allows for the determination of Drude components (very weak in this case) and the role of vibrations, overtones and combinations. In the present case, the main issue is related to the UV range, where film quality and thickness homogeneity is essential for IVPs. Indeed, casting very thin films of high optical quality for transmittance requires very good processing (i.e. solubility) so far not available with such materials thus preventing a detailed analysis of the optical properties (in particular for the imaginary part). As a consequence, thickness fitting and real part values suffer severe uncertainty. On the other hand, we could work on bulk pressed materials (the once used to prepare gratings and Fresnel lenses). Bulk samples are for sure suitable for ellipsometry and reflectance, but useless for transmittance. Moreover, bulk materials and spin-cast films might have different density, morphology and chain orientation, all factors affecting the optical functions. At the end of the story, the accuracy of our current measurements is still not satisfactory, and we are still not able to carefully disentangle the role of the different cross-linkers used. For this reason, results previously achieved^[S3,S4]^ can be used as indicative of the current refractive index value with a possible change due to the crosslinkers roughly of the order of +/- 5%.





**Figure S17.** Reflectance spectra over different positions for a (**S-DIDBT** 50/50:PS)-CA DBR grown (2.5 bilayers; 150 rps spinning rate; 100 µL volume deposition) with low amount of IVP (<<10 mg mL^-1^) hosted in a PS matrix (25 mg mL^-1^).

**Bibliography**

1. Optical constants of highly stretch-oriented poly(p-phenylene-vinylene): A joint experimental and theoretical study, D. Comoretto, G. Dellepiane, F. Marabelli, J. Cornil, D. A. dos Santos, J. L. Brédas, D. Moses, *Phys. Rev. B* (2000): *62*, 10173-10184. <https://doi.org/10.1103/PhysRevB.62.10173>
2. Highly oriented poly(paraphenylene vinylene): Polarized optical spectroscopy under pressure, V. Morandi, M. Galli, F. Marabelli, D. Comoretto, *Phys. Rev. B* (2009): *79*, 045202. <https://doi.org/10.1103/PhysRevB.79.045202>
3. High Refractive Index Inverse Vulcanized Polymers for Organic Photonic Crystals, C. Tavella, P. Lova, M. Marsotto, G. Luciano, M. Patrini, P. Stagnaro, D. Comoretto, *Crystals* (2020): *10*, 154. <https://doi.org/10.3390/cryst10030154>
4. C. Tavella, G. Luciano, P. Lova, M. Patrini, C. D'Arrigo, D. Comoretto, P. Stagnaro, “2,5-Diisopropenylthiophene by Suzuki-Miyaura cross-coupling reaction and its exploitation in inverse vulcanization: a case study”, *RSC Adv.* (2022): *12*, 8924–8935. https://doi.org/10.1039/d2ra00654e
